# Neuropathogenicity of Two Saffold Virus Type 3 Isolates in Mouse Models

**DOI:** 10.1371/journal.pone.0148184

**Published:** 2016-02-01

**Authors:** Osamu Kotani, Asif Naeem, Tadaki Suzuki, Naoko Iwata-Yoshikawa, Yuko Sato, Noriko Nakajima, Takushi Hosomi, Hiroyuki Tsukagoshi, Kunihisa Kozawa, Hideki Hasegawa, Fumihiro Taguchi, Hiroyuki Shimizu, Noriyo Nagata

**Affiliations:** 1 Department of Pathology, National Institute of Infectious Diseases, Tokyo, Japan; 2 Department of Virology and Viral Infections, Faculty of Veterinary Medicine, Nippon Veterinary and Life Science University, Tokyo, Japan; 3 Department of Virology II, National Institute of Infectious Diseases, Tokyo, Japan; 4 The Public Health Institute of Kochi Prefecture, Kochi, Japan; 5 Gunma Prefectural Institute of Public Health and Environmental Sciences, Gunma, Japan; University of Utah, UNITED STATES

## Abstract

**Objective:**

Saffold virus (SAFV), a picornavirus, is occasionally detected in children with acute flaccid paralysis, meningitis, and cerebellitis; however, the neuropathogenicity of SAFV remains undetermined.

**Methods:**

The virulence of two clinical isolates of SAFV type 3 (SAFV-3) obtained from a patient with aseptic meningitis (AM strain) and acute upper respiratory inflammation (UR strain) was analyzed in neonatal and young mice utilizing virological, pathological, and immunological methods.

**Results:**

The polyproteins of the strains differed in eight amino acids. Both clinical isolates were infective, exhibited neurotropism, and were mildly neurovirulent in neonatal ddY mice. Both strains pathologically infected neural progenitor cells and glial cells, but not large neurons, with the UR strain also infecting epithelial cells. UR infection resulted in longer inflammation in the brain and spinal cord because of demyelination, while the AM strain showed more infectivity in the cerebellum in neonatal ddY mice. Additionally, young BALB/c mice seroconverted following mucosal inoculation with the UR, but not the AM, strain.

**Conclusions:**

Both SAFV-3 isolates had neurotropism and mild neurovirulence but showed different cell tropisms in both neonatal and young mouse models. This animal model has the potential to recapitulate the potential neuropathogenicity of SAFV-3.

## Introduction

Saffold virus (SAFV) is a cardiovirus belonging to the family *Picornaviridae*. In 2007, SAFV was isolated from a stool sample obtained from an infant who suffered a fever of unknown origin in 1981 [1]. Prior to this, cardioviruses were thought to cause serious diseases, such as myocarditis, encephalomyelitis, and diabetes, mainly in small rodents [2–6]. The zoonotic *Encephalomyocarditis virus* (which belongs to the genus *Cardiovirus*) is infectious in primates, including humans, but causes only occasional and mild outbreaks [7–12]. After its initial identification, SAFV was primarily detected in fecal and throat swab specimens from infants and children with acute gastroenteritis or acute upper respiratory symptoms [13–25]. Epidemiological and genetic analyses suggest that SAFV, especially genotypes 2 and 3, is circulating freely in the human population and is transmitted from human to human during early childhood because the sera of children and the elderly have high rates of neutralizing antibodies against SAFV genotypes 2 and 3 [18, 19, 22, 24, 26, 27]. The prevalence of SAFV in other mammals, including rodents, is still uncertain.

The pathogenicity of SAFV remains unclear because the virus is often detected along with enteric or respiratory viruses such as norovirus, rotavirus, bocavirus, and influenza virus [13, 14, 16, 17, 20, 21, 23, 25, 28–31], and even in healthy individuals [15]; thus it is difficult to attribute infections to a specific virus. On the other hand, SAFV mono-infection was found in children with diarrhea, hand-foot-mouth disease, and upper respiratory inflammation [25, 30–32]. In addition, a surveillance program for acute flaccid paralysis (for global polio eradication) conducted by the World Health Organization occasionally detected SAFV in fecal specimens from patients with non-polio acute flaccid paralysis [15, 27, 33, 34]. Although SAFV is also found in the cerebrospinal fluid (CSF) of infants with cerebellitis [35] and aseptic meningitis [36], it is rarely observed in CSF samples from those with neurological diseases [13, 30, 35]; thus the role of SAFV in neurological diseases remains uncertain.

Enteroviruses belonging to the family *Picornaviridae*, especially coxsackieviruses A and B, preferentially infect neonatal mice rather than adult mice [37]. Thus, neonatal mice have been used for viral isolation and to investigate the pathogenesis of enteroviruses. However, picornaviruses generally show high host specificity; the natural host for all human enteroviruses is the human [37]. Enterovirus 71, a member of the family *Picornaviridae*, can cause meningitis and encephalitis because it infects human neurons. Some human isolates induce paralysis in neonatal mice after experimental infection of the brain. However, the mice often show severe myositis due to viral infection of not only neurons but also skeletal muscle; this does not happen in humans [38]. By contrast, coxsackievirus B and mouse-adapted enterovirus 71 infect large neurons in the brains of neonatal or adult mice and these animal models mimic the neuropathology observed in the human brain [39–42].

Therefore, establishing a mouse model of SAFV infection would advance understanding of the neuropathogenesis of SAFV. Indeed, a recent report [43] shows that mice subjected to intracerebral inoculation with HeLa cell-adapted SAFV type 2, which had been passaged eight times in rhesus monkey kidney epithelial (LLC-MK_2_) cells and then 13 times in the alpha/beta interferon-deficient human glial cell line, U118MG, developed neurological disorders. In the present study, the primary aim was to examine the susceptibility of mouse brain to SAFV-3 in the absence of viral host adaptation. We used two clinical isolates of SAFV-3: the JPN 08–404 strain (isolated from the CSF of a patient with aseptic meningitis) [36], and the Gunma/176/2008 strain (isolated from a throat swab from a patient with acute upper respiratory inflammation) [21]. Neonatal and young ddY mice, and young BALB/c mice were infected and analyzed using virological, pathological, and immunological methods. The results showed that both clinical isolates of SAFV-3 infected neural progenitor cells and glial cells, but not large neurons, and that both were mildly neurovirulent in mice; however, the clinical isolates showed different cell tropism and neurovirulence. Taken together, these results suggest that SAFV-3 is a candidate neuropathogenic virus that causes aseptic meningitis and other neurological disorders.

## Materials and Methods

### Viruses and cells

Two clinical isolates of SAFV-3 were used in this study: JPN 08–404, obtained from the CSF of a patient with aseptic meningitis in 2008 (referred to herein as the AM strain) [36], and Gunma/176/2008, acquired from a throat swab from a patient with upper respiratory tract inflammation in 2008 (referred to herein as the UR strain) [21]. The clinical samples were obtained by the local health authorities of Kochi Prefecture (AM strain) or Gunma Prefecture (UR strain) in 2008 for the surveillance of viral diseases in Japan, but we used clinical isolates that were supplied from these local health authorities. Thus, this study did not involve research on human subjects and thus does not need the approval of the Ethical Review Board. After isolation in non-human primate LLC-MK_2_ cells (AM strain) or in human HEp-2 cells (UR strain), the stock viruses were passaged (three times for the AM strain and once for the UR strain) in LLC-MK_2_ cells cultured in minimal essential medium (MEM) supplemented with 2% fetal bovine serum, 100 U/ml penicillin, 100 μg/ml of streptomycin, and 1.5 μg/ml of Fungizone Antimycotic (Gibco, Life Technologies Corporation, Carlsbad, CA) (referred to herein as 2MEM). The virus-infected LLC-MK_2_ cells were disrupted using three freeze-thaw cycles and centrifuged at 2,000 × *g* for 15 min. The supernatant was stored at 80°C until required. Titers of the stock viruses were expressed as 50% of the cell culture infectious dose (CCID_50_)/ml in LLC-MK_2_ cells, which was calculated using the Behrens—Kärber method. All work with infectious SAFV-3 was performed under biosafety level two conditions.

### SAFV-3 UR strain genome sequencing

SAFV-3 UR strain RNA was extracted from virus-infected cell cultures using an RNeasy Plus Mini Kit (Qiagen, Hilden, Germany). Reverse transcriptase (RT)-PCR was performed with the OneStep RT-PCR Kit (Qiagen) using specific primers [27, 44]. The amplified DNA PCR products were purified using MonoFas DNA Purification Kit I (GL Sciences Inc., Tokyo, Japan) and then sequenced using an ABI 3130 Genetic Analyzer (Applied Biosystems, Life Technologies Corporation). The nucleotide sequences of the SAFV-3 UR strain were analyzed using Sequencher software (ver. 4.10.1, Gene Codes Corporation, Ann Arbor, MI). All nucleotide sequences analyzed in this study were submitted to the DNA Data Bank of Japan.

### Experimental infection of mice

Pregnant and 5-week-old female ddY mice, an outbred strain, and 5-week-old female BALB/c mice, an inbread strain, were purchased from Japan SLC (Shizuoka, Japan). The ddY strain was maintained as a closed colony and shows good reproductive performance and growth [45].

Within 24 h of birth, neonatal ddY mice were inoculated intracerebrally or intraperitoneally with the SAFV-3 AM or UR strains (10^4^ CCID_50_ in 10 μl per mouse). 2MEM was used as a negative control and as the diluent whenever needed. The mice were observed for clinical manifestations, and their body weight was measured daily for 21 days. Additional inoculated animals were sacrificed at various time points to examine virus replication and pathology (n = 3, 4, or 7 mice per group).

Six-week-old ddY and BALB/c mice (referred to hereafter as young mice) were anesthetized with isoflurane and inoculated intracerebrally with the AM or UR strains of SAFV-3 (10^4^ CCID_50_ in 50 μl). The mice were monitored for clinical signs of infection, and body weight changes were measured for 8 (ddY) or 60 (BALB/c) days. The mice were sacrificed under excess isoflurane anesthesia on 3, 8, 21, or 60 days post-inoculation (p.i.) and subjected to pathological analysis (n = 3–6 per group). The young ddY and BALB/c mice used as negative controls were inoculated intracerebrally with 2MEM.

Young BALB/c mice (n = 10 or 13 mice per group) were inoculated intracerebrally (10^4^ CCID_50_ in 50 μl per mouse), intraperitoneally (10^4^ CCID_50_ in 100 μl), intravenously (10^4^ CCID_50_ in 100 μl), intranasally (10^4^ CCID_50_ in 20 μl), or orally (10^4^ CCID_50_ in 100 μl containing 5% sucrose) with the AM or UR strains. 2MEM was used as a negative control and as the diluent whenever needed. Intracerebral inoculation was conducted under isoflurane anesthesia, and intranasal inoculation was performed under a mixture of ketamine and xylazine anesthesia [46]. Before oral inoculation, animals were deprived of water for 6 or more hours. Feces were obtained from orally-inoculated animals on Days 3 and 8 p.i. and used for viral isolation. All inoculated mice were observed for clinical signs of infection and body weight was measured daily for 21 days (n = 3, 4, or 5 mice per group). The mice were sacrificed under excess isoflurane anesthesia on Days 3, 8, and 21 p.i. and examined using virological and pathological methods.

Animal studies were carried out in strict accordance with the Guidelines for Proper Conduct of Animal Experiments of the Science Council of Japan. The animal experiments were conducted in strict compliance with animal husbandry and welfare regulations. All animal experiments were approved by the Committee on Experimental Animals at the National Institute of Infectious Diseases in Japan (approval No. 211028, 212031, 112075, 113090, and 114102), and all experimental animals were handled in biosafety level two animal facilities according to the guidelines of this committee. If necessary, viral inoculations were performed under anesthesia, and all efforts were made to minimize potential pain and distress. After inoculation, animals were monitored once a day during the study. The humane endpoint was used for all mice displaying the clinical diagnostic criteria of severe central nervous system (CNS) stress, such as circling, blindness, and convulsion, and more than 20% weight loss. Animals were euthanized under anesthesia if severe disease symptoms or weight loss was observed, while no animals became severely ill or moribund at any point prior to the experimental endpoint.

### Histopathology and immunohistochemistry

Neonatal mice were sacrificed by exposure to excess isoflurane and perfused with 10% phosphate buffered formalin (injected directly into the heart). Harvested tissues were immersed in a formalin solution overnight. The whole head, including the brain, was cut into sagittal sections, which were then immersed in a formalin solution for 2 or 3 days. Young mice were also sacrificed by exposure to excess isoflurane and exsanguinated (via the heart). The head, including the brain, the spine including the spinal cord, muscle, heart, lungs, liver, spleen, kidneys, pancreas, stomach, and intestine were harvested and immersed in a formalin solution for 2 or 3 days. After fixation, the tissues were dehydrated in ethanol (30% to 50% solutions) and decalcified in a buffered EDTA 2Na solution (Dojindo, Kumamoto, Japan) as required. The tissue samples were then embedded in paraffin and stained with hematoxylin and eosin (H&E). The brain and spinal cord tissues were also stained with Klüver-Barrera Luxol fast blue staining (KB-LFB).

A polymer-based detection system was used for immunohistochemical analysis. For antigen retrieval, deparaffinized sections were placed in a retrieval solution (pH 6) (Nichirei Biosciences, Inc., Tokyo, Japan) and heated to 121°C for 10 min in an autoclave. After washing in phosphate buffered saline (PBS), the sections were treated with 0.3% hydrogen peroxide in methanol for 30 min to quench endogenous peroxidase activity. After further washing, the sections were incubated at room temperature in 10% normal goat serum (Dako, Glostrup, Denmark) for 5 min, followed by an overnight incubation at 4°C with the primary anti-SAFV-3 polyclonal antibody or anti Iba1 polyclonal antibody (Wako Pure Chemical Industries, Ltd, Osaka, Japan). The SAFV-3 polyclonal antibody was used alongside serum from a rabbit hyper-immunized with the SAFV-3 AM strain [36]. After further washing in PBS, the sections were incubated with Nichirei-Histofine Simple Stain Mouse MAX PO (R) (Nichirei Biosciences) according to the manufacturer’s instructions. Peroxidase activity was detected with 3, 3’-diaminobenzidine (Sigma-Aldrich, St. Louis, MO), and the sections were counterstained with hematoxylin.

### Double immunofluorescence staining

To characterize the virus-infected cells, paraffin-embedded tissues were subjected to a double immunofluorescence staining procedure using a rabbit antiserum against SAFV-3, and neuronal markers such as mouse or rat monoclonal antibodies against Musashi-1 (clone D270-3; Medical & Biological Laboratories, Nagoya, Japan), anti-glial fibrillary acidic protein (GFAP) (clone GA5; Merck Millipore, Billerica, MA), anti-glutamate-aspartate transporter (GLAST) (clone ACSA-1; Miltenyi Biotec, Auburn, CA), anti-brain lipid binding protein (BLBP) (clone AT1D1; Abcam, Cambridge, UK), microtubule associated protein-2 (MAP-2) (clone A60; Millipore), anti-myelin 2,3-cyclic nucleotide 3-phosphodiesterase (CNPase) (SMI91; BioLegend, San Diego, CA), myelin-associated glycoprotein (MAG) (ab89780; Abcam), and anti-cytokeratin (clone AE3; Chemicon, Merck Millipore), and lectin biotinylated *GRIFFONIA SIMPLICIFOLIA* (GS-lectin) (Vector, Southfield MI). The antigens were retrieved by autoclaving the sections in retrieval solution (pH 6.0) (Nichirei Biosciences) at 121°C for 10 min. The sections were incubated with the primary monoclonal antibody (against Musashi-1, GFAP, GLAST, BLBP, MAP-2, CNPase, MAG, or cytokeratin) or GS-lectin, followed by incubation with antiserum against the SAFV-3 antigen. Goat anti-rabbit Alexa Fluor 568 (to detect viral antigen-binding sites), goat anti-rat Alexa Fluor 488 (to detect antibodies bound to Musashi-1), goat anti-mouse Alexa Fluor 488 (to detect antibodies bound to GFAP, GLAST, BLBP, MAP-2, CNPase, MAG, or cytokeratin) and streptavidin, Alexa Fluor 488 conjugate (to detect GS-lectin) were used as secondary antibodies (all secondary antibodies were obtained from Molecular Probes, Life Technologies Corporation). The sections were incubated with the antibodies for 30 min at room temperature before being mounted in SlowFade Gold antifade reagent containing 4', 6-diamidino-2-phenylindole (DAPI; Molecular Probes, Life Technologies Corporation). Fluorescence images were captured under a laser scanning confocal microscope (FV1000-D, Olympus, Tokyo, Japan).

### RNA *in situ* hybridization

Viral RNA was detected in sections of paraffin-embedded mouse brain by RNA *in situ* hybridization using the QuantiGene ViewRNA ISH Tissue Assay (Affymetrix, Santa Clara, CA) according to the manufacturer’s instructions, with some modifications [47]. The QuantiGene ViewRNA Probes Sets were designed using the whole gene for the SAFV-3 AM strain (accession no. HQ902242). Prior to RNA *in situ* hybridization, total RNA was extracted using a PureLink FFPE RNA Isolation Kit (Invitrogen, Life Technologies Corporation) to determine whether the viral RNA was preserved in the paraffin-embedded specimens. The RNA was then treated with the reagents provided in the TURBO DNA-free Kit (Ambion, Life Technologies Corporation) to remove contaminating DNA. RNA-extracted samples were subjected to real-time PCR to detect SAFV-3, and appropriate samples were selected for RNA *in situ* hybridization. After deparaffinization, the sections were boiled with pretreatment solution for 10 min and treated with protease QF at 40°C for 10 min. The sections were incubated with the QuantiGene ViewRNA Probe Set at 40°C for 2 h and then hybridized with PreAmplifier Mix QT at 40°C for 25 min. After treatment with Amplifier Mix QT at 40°C for 15 min, the sections were reacted with the labeled probe conjugated to alkaline phosphatase. After receiving alkaline phosphatase enhancer treatment, the sections were stained using a Warp Red Chromogen Kit (Biocare Medical, Concord, CA).

### Reverse transcriptase PCR (RT-PCR) and real-time RT-PCR of the SAFV-3 genome

Neonatal mice were sacrificed by exposure to excess isoflurane, and blood was obtained by cardiac puncture. Total RNA was extracted using the TRIzol Plus RNA Purification Kit (Ambion, Life Technologies Corporation). The brains from the ddY neonatal mice were removed from the skull, washed three times in PBS, and then separated into two parts (the cerebellum and the remaining brain, including the cerebrum and brain stem). The brains from young BALB/c mice were divided into three parts (the cerebrum, brain stem, and cerebellum) to quantify the viral RNA genome. Tissue samples (except samples of neonatal mice cerebellum) were homogenized using Lysing Matrix A (MP Biomedicals, Santa Ana, CA) and diluted in 2MEM to yield 10% homogenates. Samples of neonatal cerebellum were also homogenized using Lysing Matrix A (MP Biomedicals) in 1 ml of 2MEM. After centrifugation at 8,000 × *g* for 5 min, the supernatants were subjected to PCR to detect the viral genome. Total RNA was extracted from the supernatants of tissue homogenates generated from the organs of young mice and the brains of the neonatal mice using an RNeasy Plus Mini Kit (Qiagen). Contaminating DNA was removed from the extracted RNA using a TURBO DNA-free Kit (Ambion, Life Technologies Corporation). Nested RT-PCR was performed to detect the SAFV viral genome (the conserved 5' non-coding region; accession no. EF165067), and the product was amplified using OneStep RT-PCR (Qiagen) and TaKaRa Ex Taq (Takara Bio, Shiga, Japan) kits [27]. The first and second primer sets are listed in Table 1. The nested RT-PCR protocol was as follows: the first-round of the RT-PCR was conducted for 30 min at 50°C (for reverse transcription) and 15 min at 95°C (for Taq polymerase activation), followed by 35 cycles at 94°C for 30 sec, 60°C for 30 sec, and 72°C for 1 min. The second-round of PCR was conducted for 3 min at 95°C, followed by 35 cycles at 94°C for 30 sec, 60°C for 30 sec, and 72°C for 1 min.

**Table 1:** Primer and probe sequences used for nested reverse transcriptase (RT)-PCR and real-time RT-PCR.

Primer and probe	Genome sequence (5'–3')
Nested RT-PCR for Saffold virus	
Forward 1^st^ (CF188F)	CTAATCAGAGGAAAGTCAGCAT
Reverse 1^st^ (CR990R)	GACCACTTGGTTTGGAGAAGCT
Forward 2^nd^ (CF204F)	CAGCATTTTCCGGCCCAGGCTAA
Reverse 2^nd^ (CR718R)	GCTACTGTGAGGTCGCTACAGCTGT
Real-time RT-PCR for Saffold virus	
Forward	AAACCATGCCACAAACACCAT
Reverse	GCCYTGACCAACTACCCACAT
Probe (FAM-TAMRA)	CTTGCCGAYACACGTGACCCACA
Real-time RT-PCR for murine interferon-α 4	
Forward	CAACTCTACTAGACTCATTCTGCAAT
Reverse	AGAGGAGGTTCCTGCATCACA
Probe (FAM-TAMRA)	ACCTCCATCAGCAGCTCAATGACCTCAAA
Real-time RT-PCR for murine interferon-β	
Forward	GCTCCTGGAGCAGCTGAATG
Reverse	TCCGTCATCTCCATAGGGATCT
Probe (FAM-TAMRA)	TCAACCTCACCTACAGGGCGGACTTC
Real-time RT-PCR for murine beta-actin	
Forward	ACGGCCAGGTCATCACTATTG
Reverse	CAAGAAGGAAGGCTGGAAAAGA
Probe (FAM-TAMRA)	CAACGAGCGGTTCCGATGCCC

Primer Express software (Applied Biosystems) was used to design the primers and probes for real-time RT-PCR, all of which recognized the SAFV-3 viral protein 1 (VP1) region (accession no. HQ902242). The expression of type 1 interferon (IFN) mRNA was examined using real-time RT-PCR methods designed to detect mouse IFN-α4 and IFN-β mRNA [46, 48]. Mouse beta-actin mRNA (a housekeeping gene) was also quantified. The Taqman probe and primer sets are listed in Table 1. The real-time RT-PCR conditions used were previously published by Katano *et al*. [49].

### SAFV-3 neutralization assay

Sera were collected by centrifugation and inactivated by heating at 56°C for 30 min. Sera were titrated (in duplicate) from 1:16 to 1:256 in 96-well plates and reacted with inoculated 100 CCID_50_ of SAFV-3 at 37°C for 2 h before LLC-MK_2_ cells were added to each well. Titers of neutralization antibodies were assessed against each virus, which was identical to the virus used in the experimental infection of mice. Cells were incubated at 37°C for 2 weeks, during which time they were examined twice for the presence of viral cytopathic effects. The neutralizing antibody titer was determined as the reciprocal of the highest dilution at which no cytopathic effect was observed.

### Statistical analysis

Data are expressed as the mean and standard error of the mean. Statistical analysis was performed using Graph Pad Prism 5 software (GraphPad Software Inc., La Jolla, CA). Intergroup comparisons were performed using unpaired *t*-tests or one-way analysis of variance (ANOVA), followed by Tukey’s *post* test. A *P*-value <0.05 was considered statistically significant.

## Results

### Amino acid sequence differences between the two SAFV-3 strains

The complete genomes for the AM and UR strains of SAFV-3 were compared. The nucleotide sequence for the complete genome of the AM strain (accession no. HQ902242) was 98.6% identical to that of the UR strain (accession no. AB983594). The AM strain genome differenced from the UR strain with respect to 114 nucleotides, eight of which were non-synonymous. Regarding the structural proteins, the VP2 protein of the AM and UR strains differed by only a single amino acid (at position 269: Thr in AM and Asn in UR). By contrast, the non-structural proteins showed differences at the following seven amino acid positions: position 31 (AM, Asn; UR, Asp) in the leader protein; position 113 (AM, Pro; UR, Ser) in the 2A protein; positions 31 (AM, Ala; UR, Thr) and 262 (AM, Ala; UR, Glu) in the 2C protein; position 13 (AM, Met; UR, Thr) in the 3B protein; position 112 (AM, Asn; UR, Asp) in the 3C protein; and position 387 (AM, His; UR, Tyr) in the 3D protein.

### Neurovirulence of SAFV-3 in neonatal ddY mice

**Fig 1:**
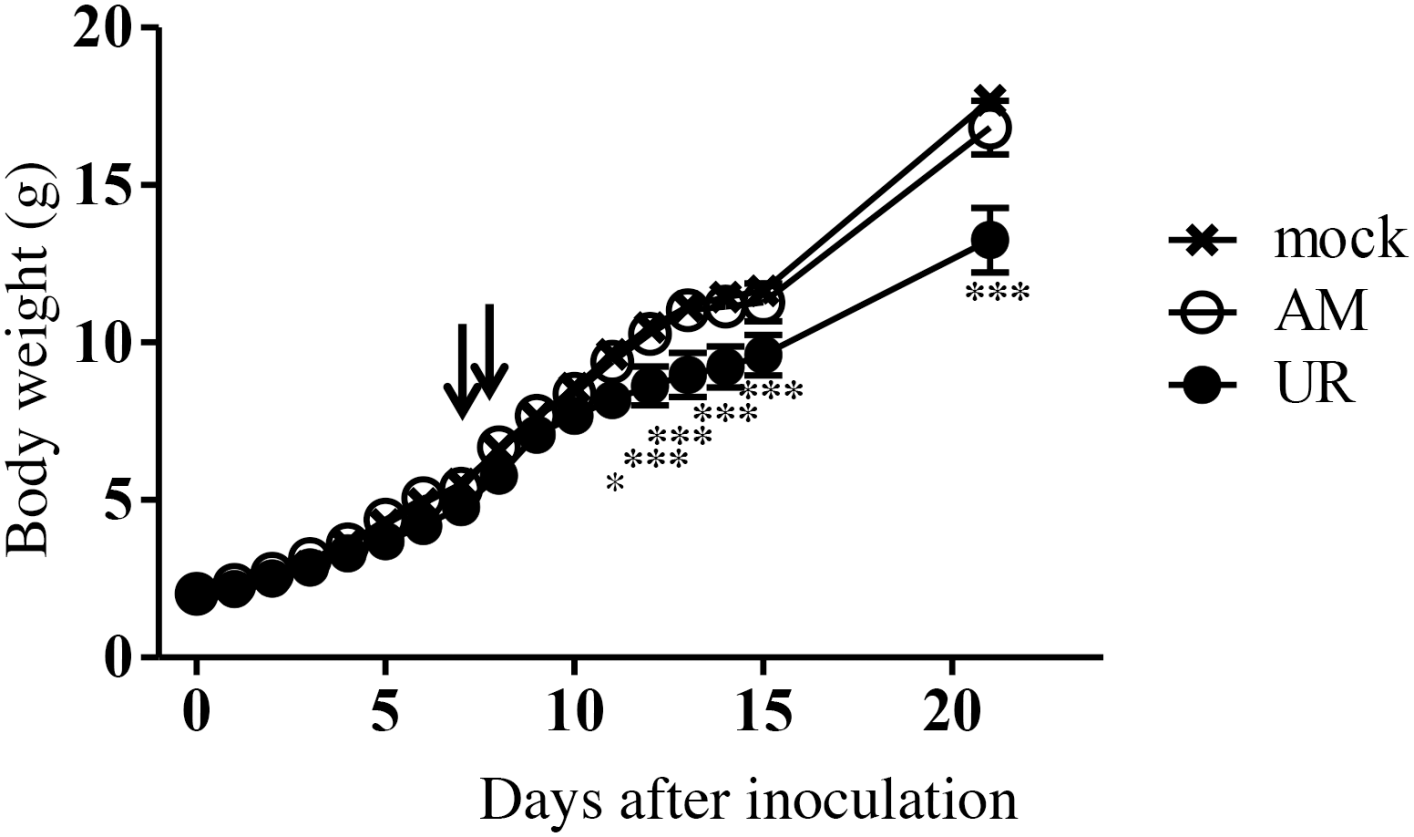
Clinical course of neonatal ddY mice after intracerebral inoculation with SAFV-3. Within 24 h of birth, neonatal ddY mice were inoculated intracerebrally with 10^4^ CCID_50_ (cell culture infectious dose) of the aseptic meningitis (AM) or upper respiratory (UR) strains of SAFV-3. Animals were observed for clinical manifestations, and body weight was measured daily for 15 days and on Day 21 (n = 3–4 mice per group). Two of the four AM-inoculated mice showed mild neurological signs, such as rolling and ataxia, on Days 7 and 8 post-inoculation (p.i.) (arrows), but the animals rapidly recovered. UR-inoculated mice showed significantly less weight gain than mock-infected control mice. All animals survived the inoculation. (*P < 0.05 and ***P < 0.001; one-way ANOVA). The data in the figure are representative of two experiments with similar results.

To investigate the neurovirulence of SAFV-3, neonatal ddY mice were inoculated intracerebrally with the AM or UR strain of SAFV-3. We found that 50% of the AM-inoculated neonatal mice developed mild neurological signs, such as elevated tails, ataxia, and rolling, on Days 7 and 8 p.i.; however, all mice recovered rapidly. The rate of weight gained in the UR-inoculated neonatal mice was significantly lower than that in the 2MEM-inoculated control mice during the observation period, whereas that in AM-inoculated neonatal mice was equal to that in the 2MEM-inoculated control mice (Fig 1). The observed neuronal deficits suggested that viral infection might affect the cerebellum in neonatal mice, especially in those inoculated with the AM strain. This result suggested that both SAFV-3 strains were mildly virulent in neonatal mice. Additionally, the virulence of the two SAFV-3 strains differed in neonatal mice after intracerebral inoculation.

### Histopathology and cell tropism of SAFV-3 in neonatal ddY mice

**Fig 2:**
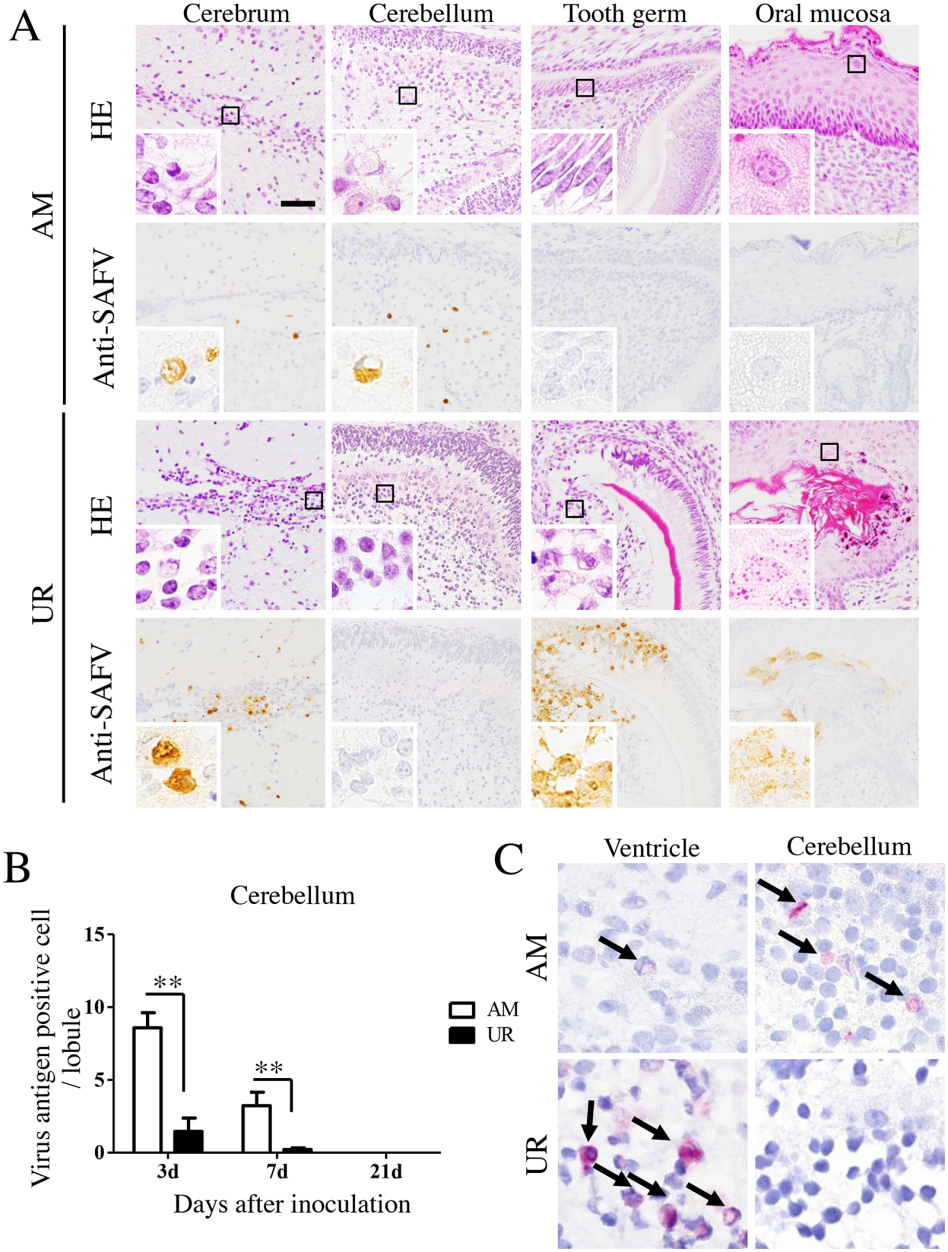
Histopathological examination of neonatal ddY mice inoculated intracerebrally with SAFV-3. Within 24 h of birth, neonatal ddY mice were inoculated intracerebrally with 10^4^ CCID_50_ (cell culture infectious dose) of the aseptic meningitis (AM) or upper respiratory (UR) strains of SAFV-3. (A) Representative histopathological findings of viral infection in neonatal mice 3 days post-inoculation (p.i.) are shown (n = 3–4 mice per group). Hematoxylin and eosin staining (H&E) and immunohistochemical analysis with an anti-SAFV-3 antibody (anti-SAFV). Bar, 50 μm. Mild histopathological changes were associated with viral antigen-positive cells (brown) in AM-inoculated neonatal mice. Degenerated and necrotic cells and a mild inflammatory infiltrate were observed around the lateral ventricle, in the tooth germ, and in the oral mucosa of UR-inoculated neonatal mice. Viral antigen-positive cells were present in the lesion. The cytoplasm of degenerated cells (with condensed nuclei) in the cerebrum and tooth germ was positive for viral antigens (insets). Meanwhile, the oral mucosa of UR-inoculated mice appeared histopathologically normal, but was positive for viral antigens (inset). (B) Number of viral antigen-positive cells in the cerebellum. (**P < 0.01; unpaired *t*-test). (C) *In situ* hybridization to visualize viral RNA in the brain of a neonatal mouse on Day 3 p.i. Viral RNA positive signals (red; arrows). Viral RNA was observed in viral antigen-positive tissue. Original magnification, 400× (A), and 1,000× (insets in A and C).

We also used histopathological and immunohistochemical approaches to examine the ability of the SAFV-3 virus to infect neonatal mice after intracerebral inoculation (Table 2 and Fig 2). On Day 3 p.i., the AM-inoculated mice showed mild or no histopathological changes in the brain or other major organs, although viral antigens were detected in the brain, spinal cord, and skeletal muscle (Table 2 and Fig 2A). UR-inoculated mice showed significant histopathological changes in the brain and tooth germ, including cellular degeneration with condensed nuclei and necrosis with slight cellular infiltration (Table 2 and Fig 2A). SAFV antigens were detected in degenerated neural cells (which showed eccentric nuclei) around the lateral ventricle, which was the virus inoculation site, and in the undeveloped medulla of the cerebellum (Fig 2A, insets). In addition, a few neural cells within the spinal cord, and skeletal muscle cells throughout the body, were positive for viral antigens (Table 2). However, none of the large pyramidal neurons in the brain and spinal cord were positive for viral antigens. Moreover, viral antigens were detected in the oral cavity of UR-inoculated mice, but the histopathological changes were very weak. The number of viral antigen-positive cells in the cerebellum lobule of AM-inoculated mice was approximately five times greater than that in UR-inoculated mice on Day 3 p.i., and 16 times greater on Day 7 p.i. (Fig 2B). RNA *in situ* hybridization using paraffin-embedded brain tissues harvested from neonatal mice at 3 days p.i. revealed that viral RNA was present in the ventricle, cerebrum, and brain stem of mice inoculated with either virus strain (Fig 2C). Notably, the cerebellum in AM-inoculated mice, but not in UR-inoculated mice, was positive for viral RNA. The distribution of viral antigens correlated with that of viral RNA in the brain.

**Table 2:** Histopathological examination of viral antigen-positivity and inflammatory reactions following intracerebral inoculation of Saffold virus into neonatal ddY mice.

Virus strain	Day of sacrifice (p.i.)	Number of mice	Central nervous system tissues				Other tissues			
			Cerebrum	Brain stem	Cerebellum	Spinal cord	Muscle	Pancreas	Oral mucosa	Tooth germ
AM	3	3	3/1*	3/1	3/0	2/0	2/0	0/0	0/0	0/0
	7	3	1/0	0/0	3/0	1/0	0/0	0/0	0/0	0/0
	21	7	0/1	0/2	0/1	0/0	0/0	0/0	0/0	0/0
UR	3	4	4/4	4/3	2/0	3/0	2/0	0/0	2/1	2/2
	7	4	4/1	4/2	2/0	4/1	4/0	0/0	0/0	2/2
	21	4	0/1	2/4	0/3	1/3	0/0	0/0	0/0	0/0

*Values represent the number of animals positive for viral antigen/number among animals showing evidence of degeneration or inflammatory reactions.

AM, aseptic meningitis; p.i., post-inoculation; UR, upper respiratory.

**Fig 3:**
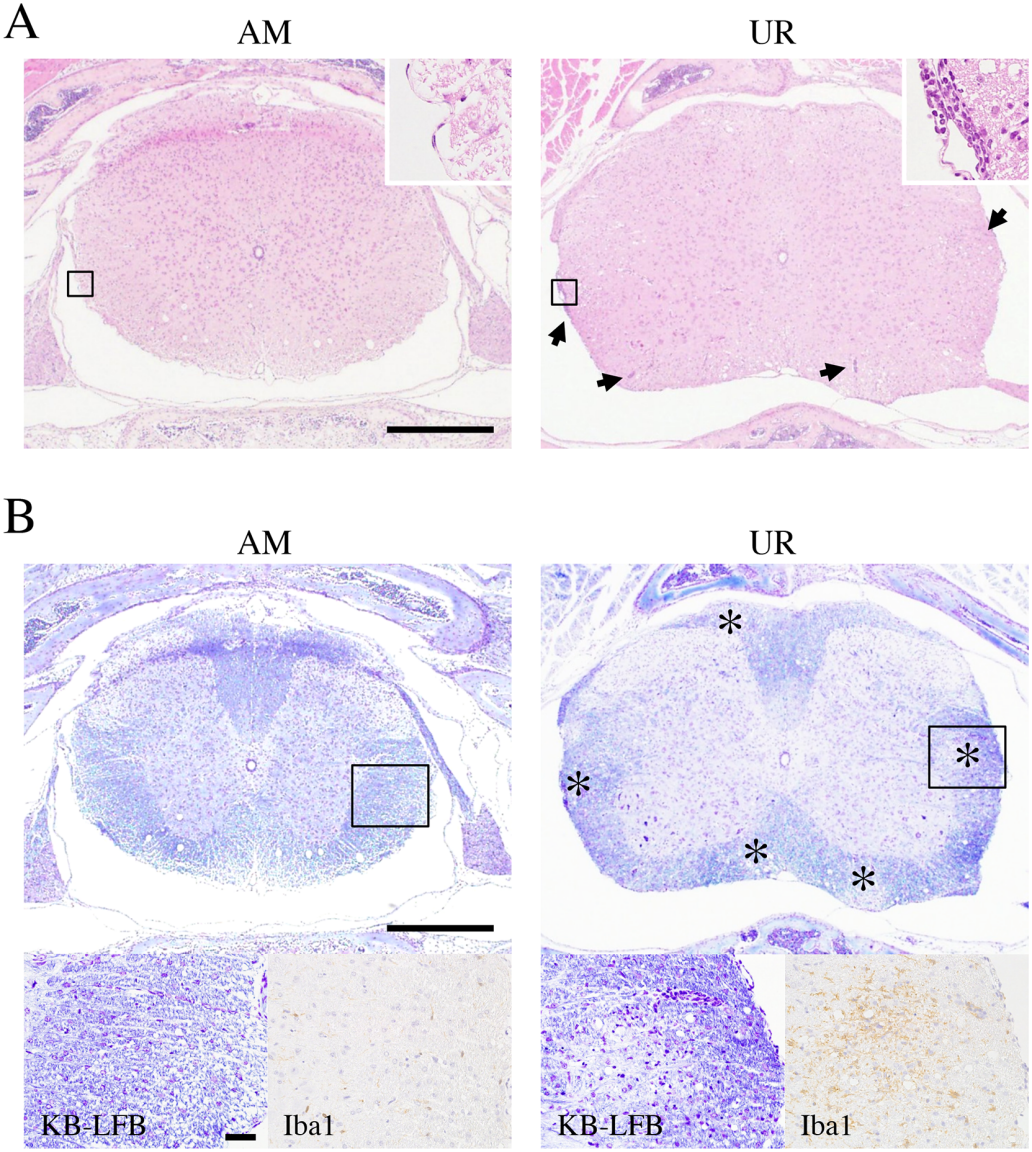
Demyelination in the spinal cord of neonatal ddY mice after intracerebral inoculation with SAFV-3. Within 24 h of birth, neonatal ddY mice were inoculated intracerebrally with 10^4^ CCID_50_ (cell culture infectious dose) of the aseptic meningitis (AM) or upper respiratory (UR) strains of SAFV-3. (A) Representative histopathological findings of the spinal cord in neonatal mice 21 days post-inoculation (p.i.) are shown. Hematoxylin and eosin staining (H&E) Bar, 500 μm. Meningitis and mild perivascular cuffing (arrows, upper right panels; box, low magnification) are observed in the white matter of the UR-inoculated mice on day 21 p.i. (B) Klüver-Barrera Luxol fast blue staining (KB-LFB) (upper panels; low magnification, lower left panels; box of upper panels) and immunohistochemical analysis with an anti-Iba1 antibody (lower right panels). Scattered demyelinations (asterisks) with inflammatory infiltrations were observed in the white matter of UR-inoculated mice. Infiltrated cells were Iba1 positive microglia (brown, lower right panels). Upper bar, 500 μm; lower bar, 50 μm. Upper panels, original magnification (50×); lower panels, 400×.

On Day 3 p.i., mild perivascular cuffing was more common in the cerebrum/brain stem of UR-inoculated mice than in that of AM-inoculated mice; however, no perivascular cuffing was observed in the cerebellum of either group (S1 Fig, upper panels). Appreciable perivascular cuffing and vacuolation in the neuropil were detected in the brains stem of UR-inoculated mice on Day 21 p.i. (S1 Fig, lower panels; Table 2), with two of the four mice showing severe focal necrosis in the thalamus. In addition, mild meningitis and perivascular cuffing was observed in the spinal cords of UR-inoculated mice but not in AM-inoculated mice (Fig 3A). Foci of demyelination with Iba1^+^ microglia infiltrations were observed in the white matter of the spinal cords of UR-inoculated mice but not of AM-inoculated mice (Fig 3B). Histopathologically, a few microglia phagocytized viral antigens in the lesion of the brainstem and spinal cord.

**Fig 4:**
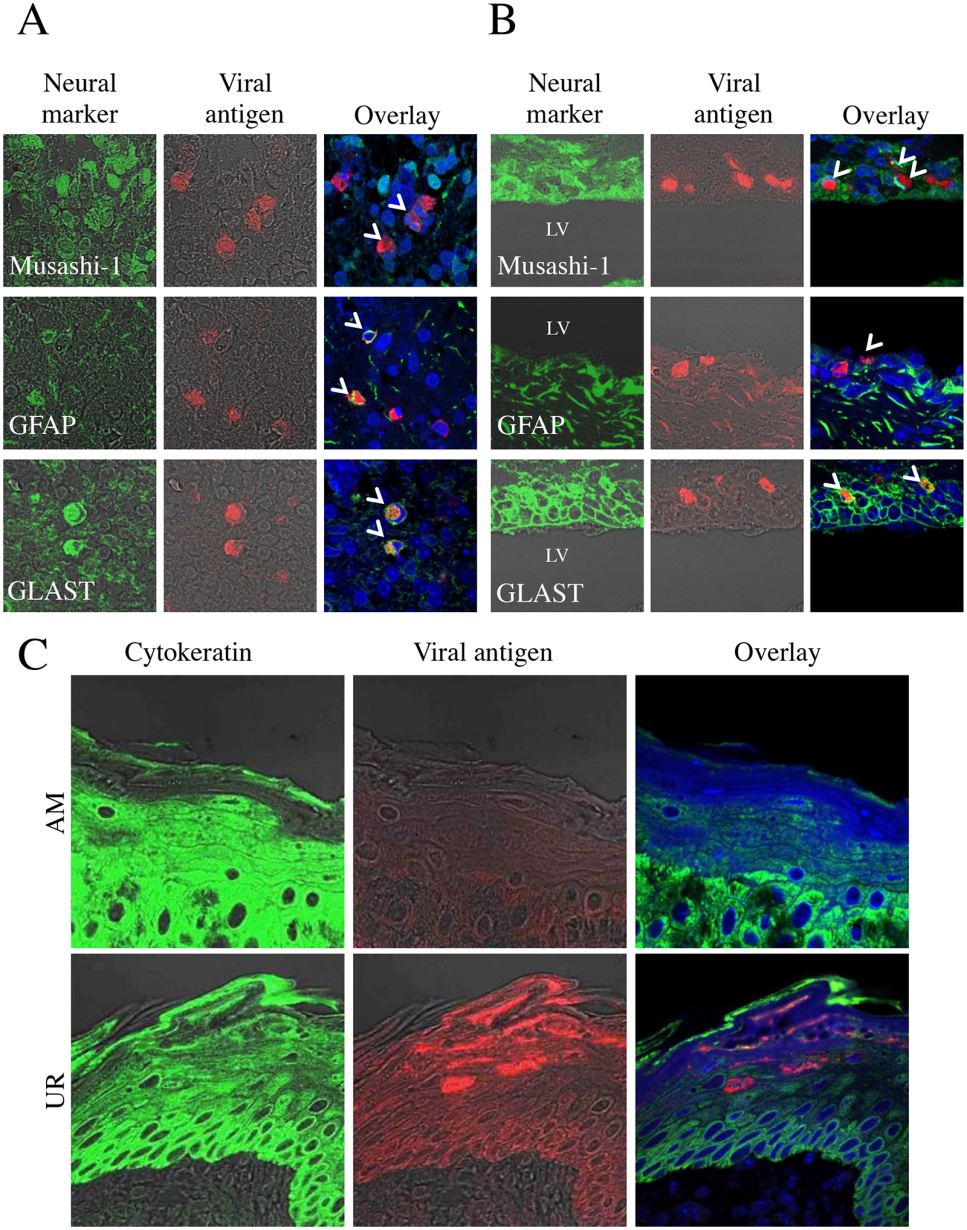
Identification of SAFV-3-infected cells in the neonatal mouse brain. Within 24 h of birth, neonatal ddY mice were inoculated intracerebrally with 10^4^ CCID_50_ (cell culture infectious dose) of the aseptic meningitis (AM) or upper respiratory (UR) strains of SAFV-3. Double immunofluorescent images showing viral antigens (red) and markers (green) for Musashi-1^+^ neural progenitor cells, GFAP^+^ astrocytes, and GLAST^+^ radial astrocytes, in the brains of mice on Day 3 post-inoculation (p.i.). (A) Viral antigen-positive cells in the cerebellum of AM-inoculated mice were identified as Musashi-1^+^ neural progenitor cells, and GFAP^+^ and GLAST^+^ radial astrocytes (aka Bergmann glia) (B) By contrast, Musashi-1^+^ neural progenitor cells (aka neuroepithelial cells), and GFAP^+^ and GLAST^+^ radial astrocytes in the ventricular zone of UR-inoculated mice were positive for viral antigens. Bar, 20 μm. (C) Viral antigen-positive cells in the oral mucosa of UR-inoculated mice were cytokeratin-positive epithelial cells. Arrows, viral antigen-positive and neural marker-positive cells (A, B). LV, lateral ventricle (B). Original magnification, 600×.

Double immunofluorescence staining indicated that GLAST^+^ or GFAP^+^ glial cells and Musashi-1^+^ neural progenitor cells in the cerebellum of AM-inoculated animals were positive for viral antigens (Fig 4A). The Musashi-1^+^ neural progenitor cells, as well as the neuroepithelial cells and GLAST^+^ or GFAP^+^ glial cells around the ventricle, were also positive in UR-inoculated mice (Fig 4B). BLBP^+^ radial astroglia cells both in the AM- and in the UR-inoculated mice were positive for viral antigens, but MAP-2^+^ neuronal cells, CNPase^+^ or MAG^+^ oligodendroglia cells, and GS-lectin^+^ microglia cells were negative for viral antigens. In addition, the cytokeratin-positive epithelial cells in the oral cavity of UR-inoculated mice were positive for viral antigens (Fig 4C), as were the calbindin-positive cells in the tooth germ [50]. The skeletal muscle cells in the tongue and the cardiac muscle cells of mice inoculated with either the AM or UR strain were positive for viral antigens (S2 Fig). However, no viral antigens were detected in other major organs, including the lungs, liver, spleen, kidneys, pancreas, stomach, and intestine, in either group of mice.

These results suggested that although both SAFV-3 isolates show a common cellular tropism in neonatal mice (infecting either glial or skeletal muscle cells), only the UR strain infected both glial and epithelial cells. However, histopathological examination suggested that the AM strain replicated in the cerebellum (Fig 2B and 2C) to a greater extent than the UR strain after intracerebral inoculation.

### Viral replication and immune responses against SAFV-3 in neonatal ddY mice

**Fig 5:**
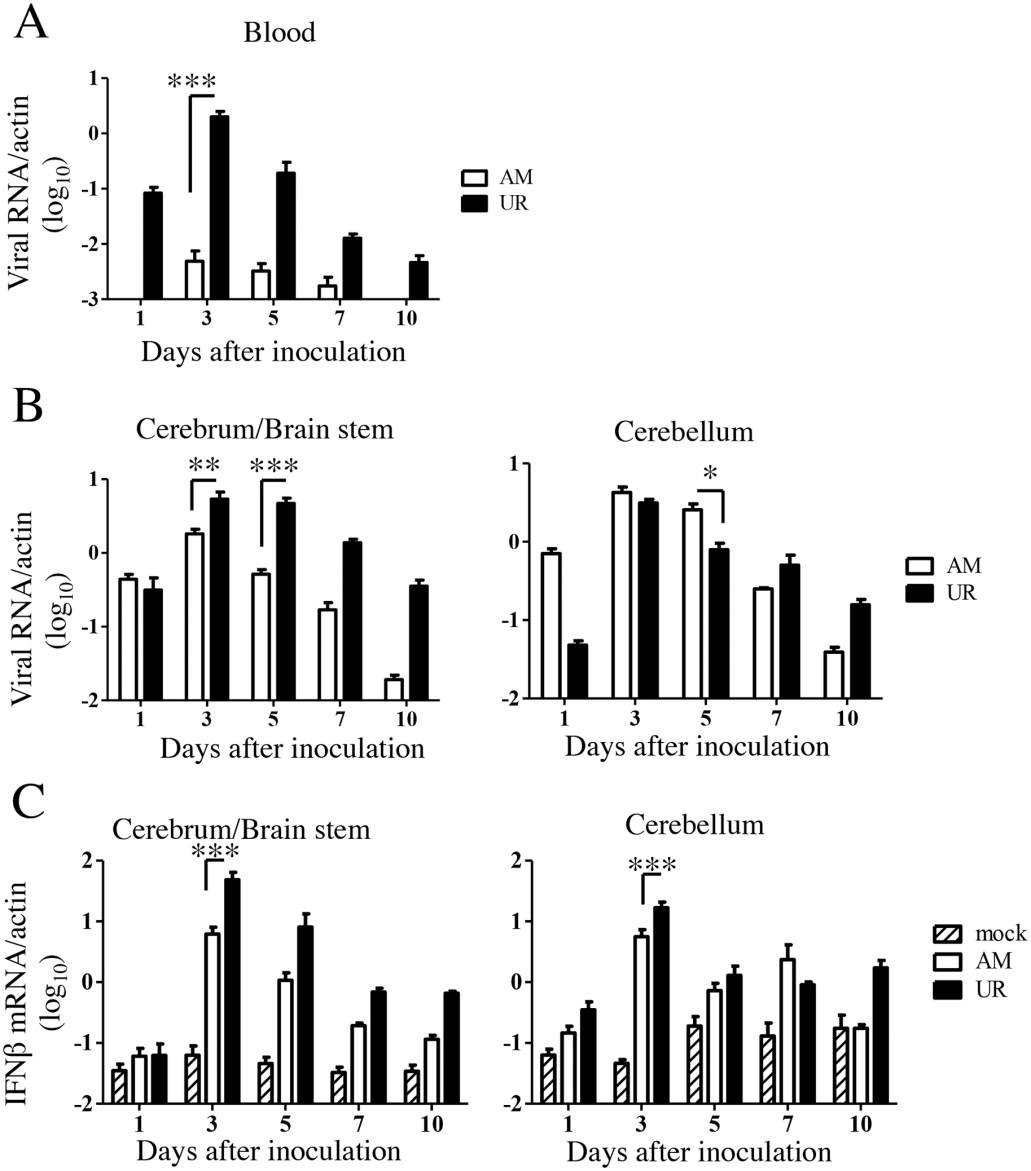
Viral replication and type 1 interferon expression in SAFV-3-inoculated neonatal ddY mice. Within 24 h of birth, neonatal ddY mice were inoculated intracerebrally with 10^4^ CCID_50_ (cell culture infectious dose) of the aseptic meningitis (AM) or upper respiratory (UR) strains of SAFV-3. The number of viral RNA copies was measured in the blood (A) and cerebrum/brain stem and cerebellum (B) of each mouse on Days 1, 3, 5, 7, and 10 post-inoculation (p.i.) (n = 4 mice per group for each day). The number of viral RNA copies is expressed relative to the number of mouse beta-actin RNA copies. Viral RNA levels in the blood and brain peaked at 3 to 5 Days p.i. in mice inoculated with AM and UR strains (*P < 0.05, **P < 0.01, and ***P < 0.001; unpaired *t*-test). (C) Real-time reverse transcriptase (RT)-PCR quantification of interferon beta (IFN-β) mRNA expression in the brains of SAFV-inoculated neonatal mice (n = 3–4 mice per group). The number of IFN-β RNA copies is expressed relative to the number of mouse beta-actin RNA copies. On Day 3 p.i., UR-inoculated mice showed greater expression of IFN-β than AM-inoculated mice (*P < 0.05 and ***P < 0.001; one-way ANOVA).

Histopathological examination and the observed neuronal deficits suggested that viremia might occur after virus inoculation, particularly in mice inoculated with the UR strain, and that viral infection might affect the cerebellum in neonatal mice, particularly those inoculated with the AM strain. Thus, viral replication was measured separately in the blood, cerebellum, and other brain regions (cerebrum and brain stem, referred to hereafter as the cerebrum/brain stem). Real-time PCR revealed that viral RNA levels in the blood of SAFV-3-inoculated mice peaked at 3–5 days p.i., although the levels were higher in UR-inoculated mice (Fig 5A). Thus, viremia was detected in both groups post-inoculation, although the levels were different. The levels of viral RNA in the cerebrum/brain stem and cerebellum of both groups increased after Day 3 p.i. Notably, on Days 3 and 5 p.i., the levels of viral RNA in the cerebrum/brain stem of mice infected with the UR strain were significantly higher than those in mice infected with the AM strain. By contrast, on Day 5 p.i., the levels of viral RNA in the cerebellum of mice infected with the AM strain were significantly higher than those in the cerebellum of mice infected with the UR strain (Fig 5B).

We next examined the anti-viral responses to SAFV infection in the brains of neonatal mice. IFN-β expression in the brains of both groups increased at 3 days p.i.; however, levels in the brains of UR-inoculated mice were significantly higher than those in the brains of AM-inoculated mice (Fig 5C). Thus, intracerebral inoculation of SAFV-3 induced an anti-viral immune response (type 1 IFN expression) in the neonatal mouse brain. In addition, the UR strain induced a stronger type 1 IFN response and longer-term inflammatory infiltration than the AM strain.

Taken together, these results suggest that both SAFV-3 strains infect the neonatal mouse brain. Additionally, the two SAFV-3 strains showed differing infectivity and virulence after intracerebral inoculation into neonatal mice.

### Neurotropism of SAFV-3 in neonatal ddY mice

We also assessed the neurotropism of SAFV-3 after intraperitoneal inoculation into neonatal ddY mice. The mice showed no obvious clinical signs after intraperitoneal inoculation with either the AM or UR strain of SAFV-3. However, histopathological and immunohistochemical examination revealed that these strains invaded and infected the CNS on Days 3 and 7 p.i. (S1 Table and S3A Fig). The UR-inoculated mouse brain contained a higher number of viral antigen-positive cells than the AM-inoculated mice (S3A Fig). In addition, viral antigens were detected in the muscle (skeletal muscle and cardiac muscle) (in two of four AM-inoculated mice and all UR-inoculated mice), tooth germ (in one of four UR-inoculated mice), and peripheral nerves of the spinal cord (in all UR-inoculated mice) on Day 3 p.i. The viral antigen-positive cells were determined morphologically to be glial cells, ependymal cells, skeletal muscle cells, or epithelial cells. An inflammatory infiltrate was observed in the CNS of UR-inoculated mice on Day 21 p.i. (S3B Fig). Double immunofluorescence staining revealed that the viral antigen-positive cells were GLAST^+^ or GFAP^+^ glial cells and Musashi-1^+^ neural progenitor cells from around the ventricle of the brain stem and the cerebellum in both AM- and UR-inoculated mice (S4 Fig). Thus, both SAFV-3 isolates showed neurotropism following intraperitoneal inoculation into neonatal mice. Although the route of infection was unclear, the UR strain showed a greater capacity to infect the CNS than the AM strain after intraperitoneal inoculation.

### Neurovirulence of SAFV-3 in young ddY mice

Young ddY mice were inoculated intracerebrally with the AM or UR strains to examine the neurovirulence of SAFV-3. At Day 6 p.i., the young UR-inoculated ddY mice gained significantly less body weight than the 2MEM-inoculated controls. There was no difference in body weight between AM-inoculated and 2MEM-inoculated ddY mice. None of the mice showed obvious clinical manifestations. Both AM- and UR-inoculated young ddY mice showed histopathological evidence of perivascular inflammation and neural cell degeneration with eccentric nuclei (S5 Fig). Viral antigen-positive cells were observed on Day 3 p.i.; double immunofluorescence staining revealed that the these cells were GLAST^+^ or GFAP^+^ glial cells and Musashi-1^+^ progenitor neural cells from within the cerebral medulla and GFAP^+^ glial cells from the cerebellum cortex (S6 Fig). In addition, viral antigens were detected in the cerebellum of two of three mice in each virus-inoculated group on Day 3 p.i. However, none of the large pyramidal neurons in the CNS were positive for viral antigens. On Day 8 p.i., all mice in both virus-inoculated groups showed meningitis and perivascular cuffing in the brain and brain stem, but not in the cerebellum. No histopathological changes were noted in the oral mucosa or tooth germ in AM- and UR-inoculated mice. These results suggested that SAFV-3 was mildly neurovirulent in young ddY mice.

### Neurovirulence and neurotropism of SAFV-3 in young BALB/c mice

**Fig 6:**
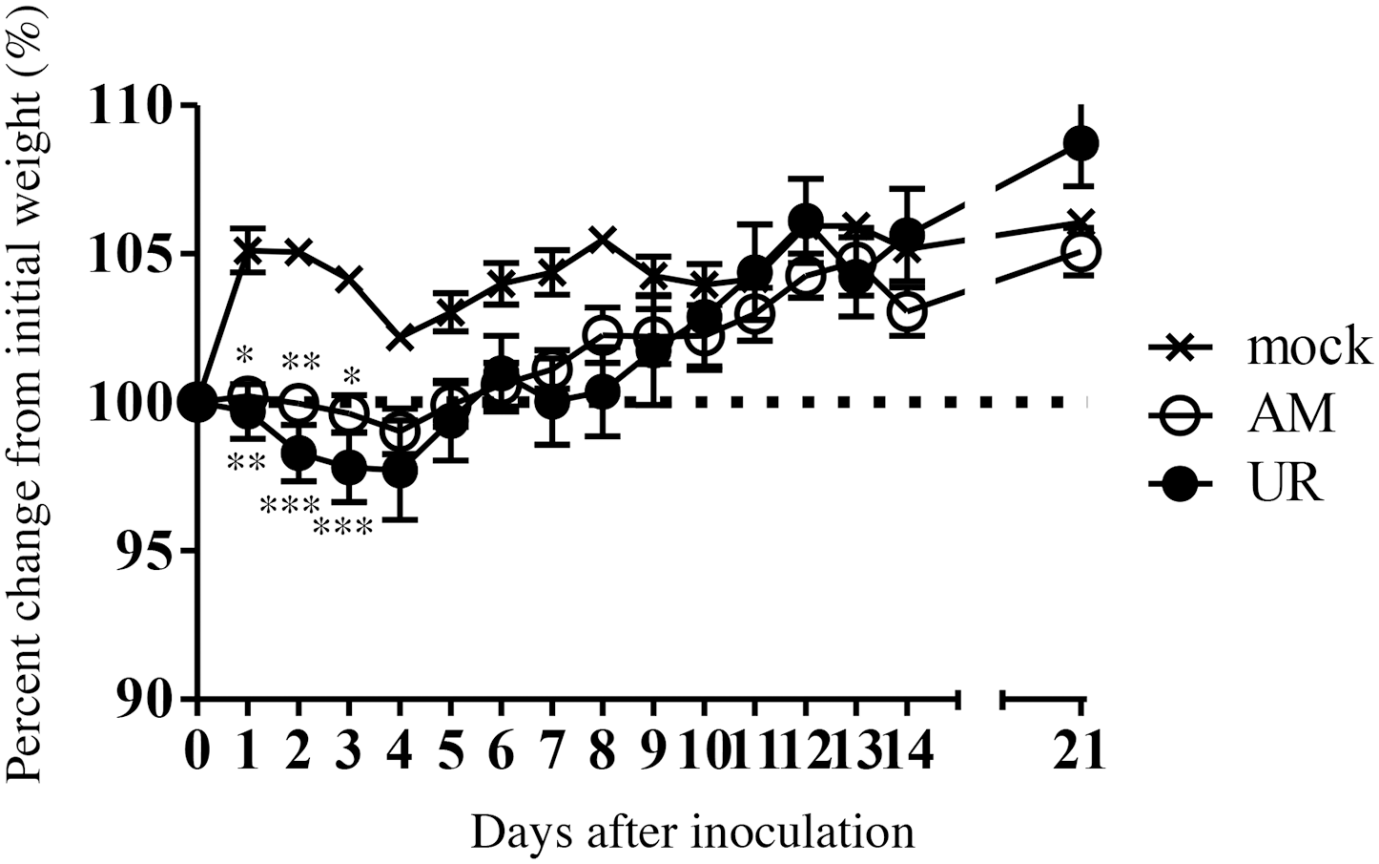
Body weight changes in young BALB/c mice after intracerebral inoculation with SAFV-3. BALB/c mice were inoculated intracerebrally with 10^4^ CCID_50_ (cell culture infectious dose) of the aseptic meningitis (AM) and upper respiratory (UR) strains of SAFV-3. Body weight was measured for 21 days post-inoculation (p.i.) (n = 6). The dotted line represents 100%. (*P < 0.05, **P < 0.01, and ***P < 0.001; unpaired *t*-test). Body weight of mice inoculated with either of the two SAFV-3 strains decreased during the early phase of infection. The figure shows representative data from two experiments with similar results.

**Fig 7:**
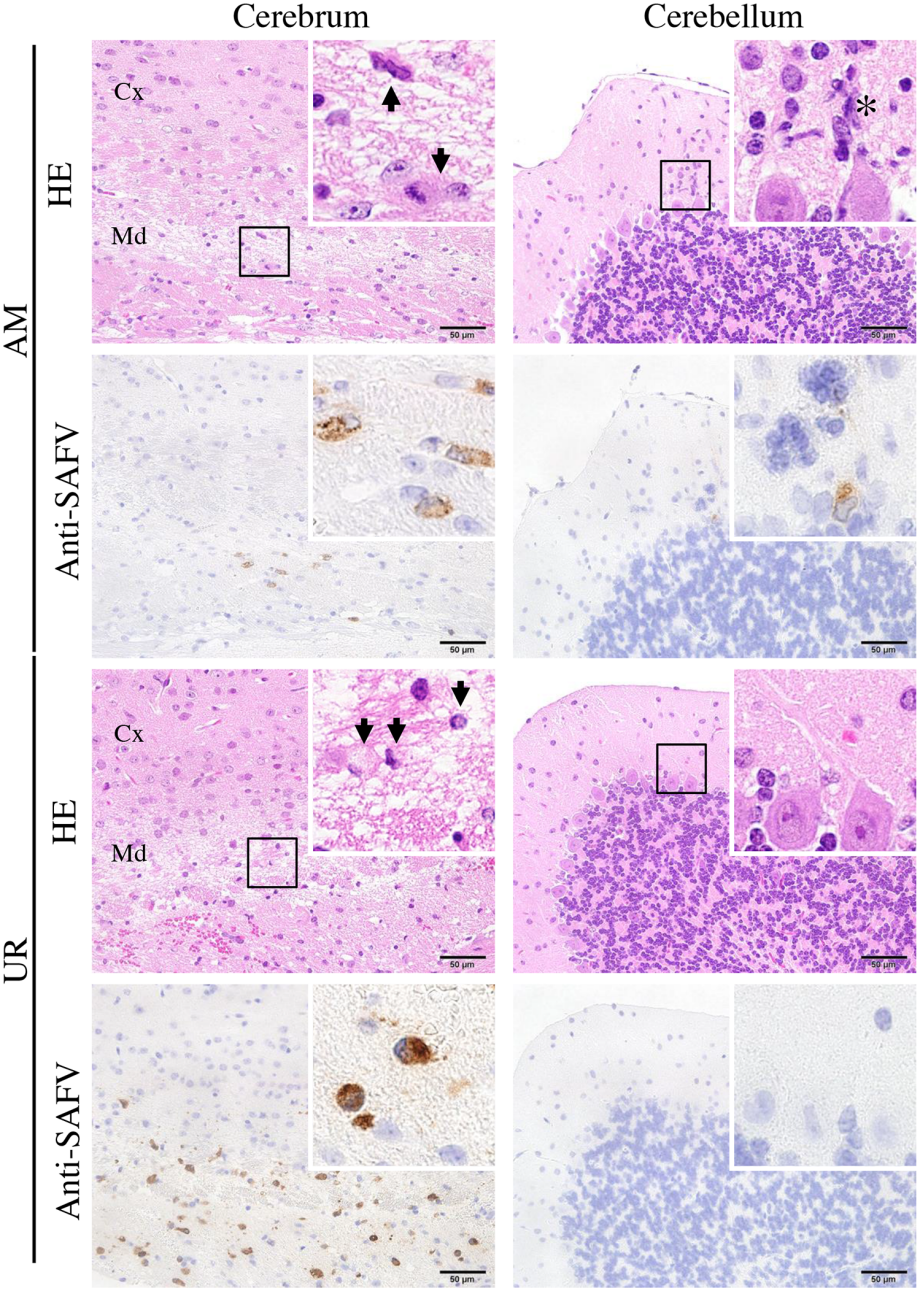
Histopathological examination of brains from young BALB/c mice after intracerebral inoculation with SAFV-3. On Day 3 post-inoculation (p.i.), brains were obtained from BALB/c mice after intracerebral inoculation with 10^4^ CCID_50_ (cell culture infectious dose) of the aseptic meningitis (AM) and upper respiratory (UR) strains of SAFV-3. Hematoxylin and eosin (H&E) staining and immunohistochemical analysis with an anti-SAFV-3 antibody (anti-SAFV). Bar, 50 μm. Nerve cells were degenerated, with eccentric nuclei (arrows), and the cerebral medulla was positive for viral antigens (insets, left panels). Several viral antigen-positive cells were seen in the lesions in both AM- and UR-inoculated mice. Nerve cells in the cerebral cortex were negative for viral antigen. Focal inflammatory infiltration (asterisk) by viral antigen-positive cells was observed in the molecular layer of the cerebellum of AM-inoculated mice, but not in that of UR-inoculated mice (insets, right panels). Purkinje cells were negative for viral antigens. Cx, Cortex; Md, Medulla. Original magnification, 400×; insets, 1,000×.

We next examined the neurovirulence and neurotropism of SAFV-3 in young BALB/c mice, which are Th-2-prone inbred mice, following intracerebral inoculation. AM- and UR-inoculated mice lost significantly more body weight than 2MEM-inoculated control mice between 1 and 3 days p.i. (Fig 6). However, mice in both groups regained the lost weight during the observation period and exhibited no obvious clinical signs. Histopathological and immunohistochemical analyses on Day 3 p.i. revealed that the degenerated and necrotic nerve cells in the brains were positive for viral antigens (Fig 7 and Table 3). All of the AM- and UR-inoculated mice showed signs of meningitis. Viral antigen-positive cells were detected in the cerebral medulla, but not in the cortex, in both groups. A greater number of viral antigen-positive cells were observed in the brains of UR-inoculated mice than in the brains of AM-inoculated mice (Fig 7). However, whereas viral antigens were detected in all of the AM-inoculated mice along with mild inflammation in the molecular layer of the cerebellar cortex on Day 3 p.i., viral antigens were detected in only two of the five UR-inoculated mice (Fig 7 and Table 3). Virus antigen-positive cells were detected in the spinal cord of a few mice on Day 3 p.i. (Table 3). The viral antigen-positive cells in the cerebral medulla of both groups were identified as GLAST^+^ or GFAP^+^ glial cells and Musashi-1^+^ progenitor neural cells by double immunofluorescence staining (S7 Fig).

**Table 3:** Histopathology after intracerebral inoculation of Saffold virus into adult BALB/c mice.

Virus strain	Day of sacrifice (i.p.)	Number of animals	Central nervous system tissues				Other tissues		
			Cerebrum	Brain stem	Cerebellum	Spinal cord	Muscle	Oral mucosa	Tooth germ
AM	3	5	5/5*	1/0	5/1	1/1	0/0	0/0	0/0
	8	4	4/4	0/1	0/4	0/3	0/0	0/0	0/0
	21	4	0/1	0/0	0/0	0/0	0/0	0/0	0/0
UR	3	5	5/4	1/2	2/0	2/0	0/0	0/0	0/0
	8	4	2/3	0/2	0/1	0/1	0/0	0/0	0/0
	21	4	0/3	0/0	0/0	0/2	0/0	0/0	0/0

*Number of animals positive for viral antigen/number of animals showing evidence of degeneration or inflammatory reactions.

AM, aseptic meningitis; p.i., post-inoculation; UR, upper respiratory

**Fig 8:**
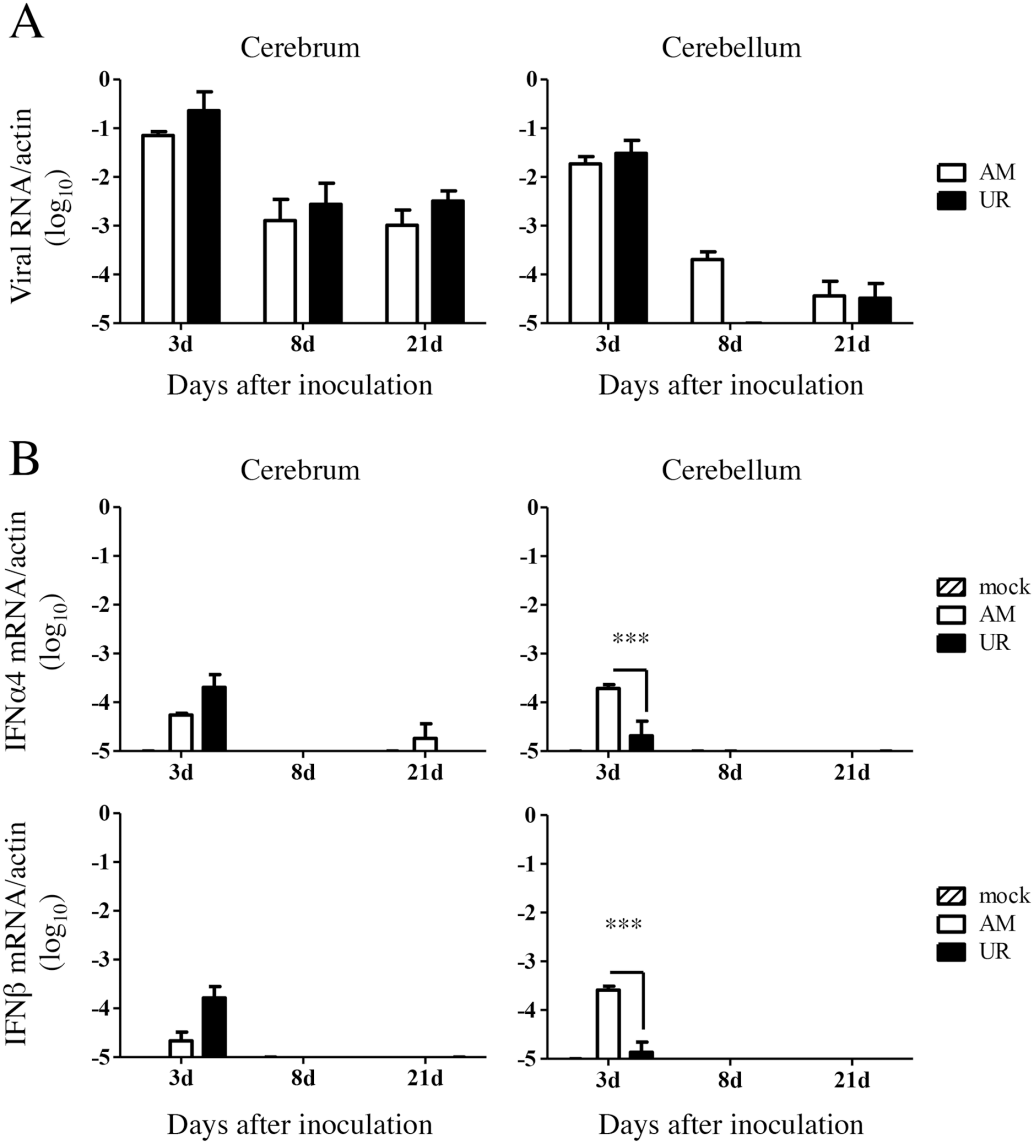
Viral replication and type 1 interferon expression in the brains of SAFV-3-inoculated young BALB/c mice. BALB/c mice were inoculated intracerebrally with 10^4^ CCID_50_ (cell culture infectious dose) of the aseptic meningitis (AM) and upper respiratory (UR) strains of SAFV-3. (A) After inoculation, the number of viral RNA copies was measured in the cerebrum and cerebellum of each mouse on Days 3, 8, and 21 p.i. (n = 3 mice per group for each day). There was no difference in the number of viral RNA copies between the AM- and UR-inoculated groups (unpaired *t*-test). (B) Quantification of type 1 interferon (IFN), IFN-α4, and IFN-β mRNA expression in the brains of SAFV-3-inoculated mice on Days 3, 8, and 21 p.i. (n = 3 mice per group for each day). Number of viral IFN-α4 and IFN-β RNA copies relative to that of mouse beta-actin copies (***P < 0.001; one-way ANOVA). On Day 3, the expression of IFN-α4 and IFN-β in the cerebellum of AM-inoculated mice was higher than that in UR-inoculated mice.

Viral RNA was detectable in the brains of mice in both groups on Days 3, 8, and 21 p.i., although at very low levels (Fig 8A). The expression of IFN α4 and β mRNA in the cerebrum of UR-inoculated mice was higher than that in AM-inoculated mice on Day 3 p.i. (Fig 8B); however, the number of IFN α4 and β mRNA copies in the cerebellum of AM-inoculated mice was significantly higher than that in the cerebellum of UR-inoculated mice (Fig 8B).

**Fig 9:**
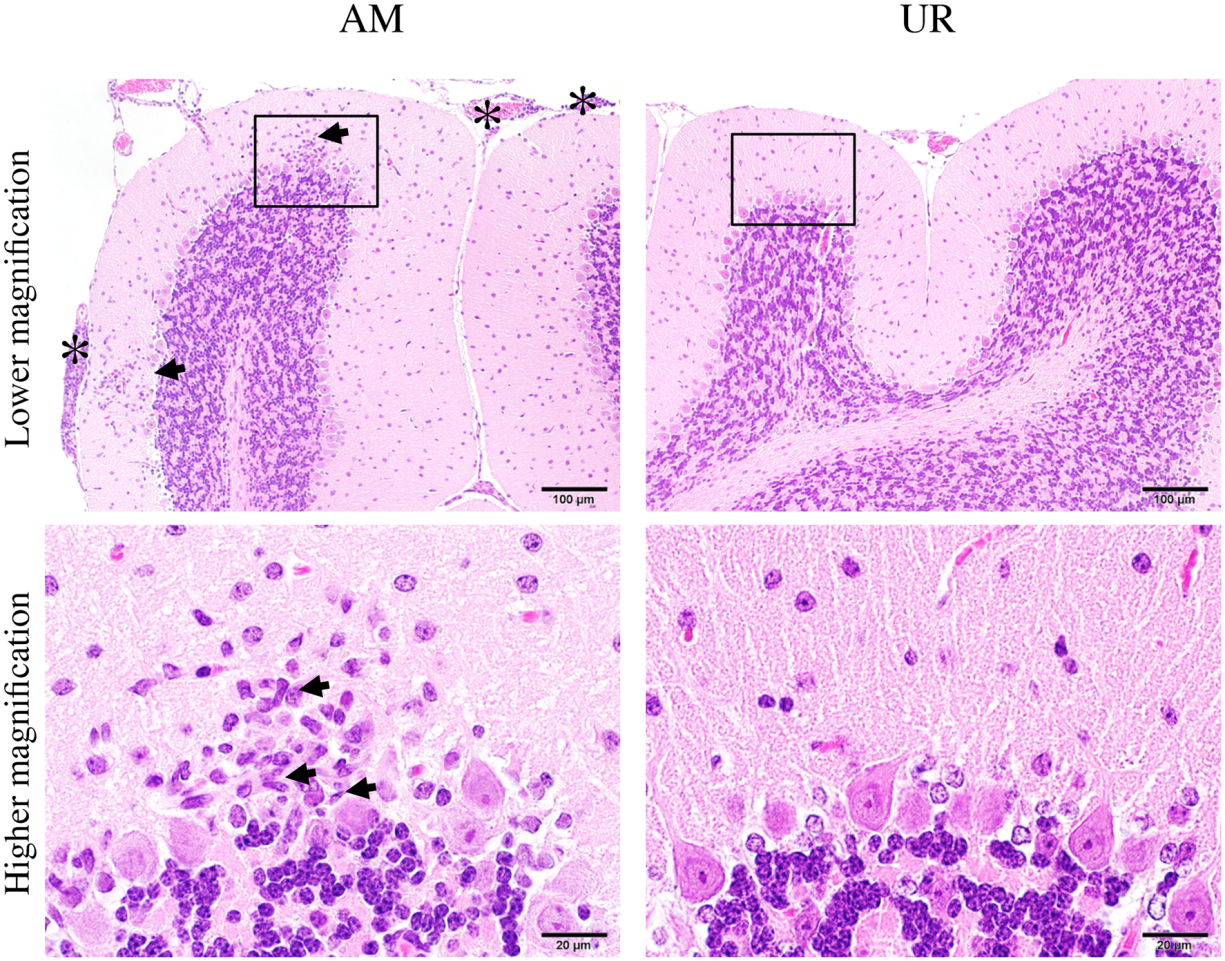
Inflammatory infiltration in the cerebellum of SAFV-3-inoculated young BALB/c mice. BALB/c mice were inoculated intracerebrally with 10^4^ CCID_50_ (cell culture infectious dose) of the aseptic meningitis (AM) and upper respiratory (UR) strains of Saffold virus type 3 (SAFV-3). Hematoxylin and eosin (H&E) staining. Bar, 100 μm (upper panels) and 20 μm (lower panels). Histopathological findings in the cerebellum on Day 8 p.i. Inflammatory cells were observed in the cerebellar cortex (arrows, low magnification) and meninges (asterisk) of AM-inoculated mice (left panels), but not in those of UR-inoculated mice (right panels). Mononuclear cells and rod-shaped cells (arrows, high magnification) were observed in the cortex and meninges of AM-inoculated, but not UR-inoculated, mice (lower panels). Purkinje cells were present in the lesion. The figure shows representative data from two experiments with similar results. Original magnification: upper panels, 200×, and lower panels, 1,000×.

Histopathologically, inflammatory infiltrations were detected in the cerebrum of AM- and UR-inoculated mice on Day 3 p.i. Although no type 1 IFN was expressed on Day 8 p.i., inflammatory infiltration was observed in the cerebellum of all AM-inoculated mice, whereas it was observed only in the cerebellum of one UR-inoculated mice (Fig 9). On Day 21 p.i., mild perivascular cuffing was observed in the spinal cord of two of the four UR-inoculated mice. None of the mice displayed histopathological changes in muscle, oral mucosa, or tooth germ (Table 3). On Day 60 p.i., neither AM- nor UR-inoculated mice had viral antigen-positive cells in the brain. However, they were positive for viral RNA; one of the six brains from AM-inoculated mice was positive and three of six brains from UR-inoculated mice were positive in the second round PCR of nested RT-PCR for SAFV (S2 Table). However, viral RNA was not detected in the paraffin embedded brain tissues of either AM- or UR-inoculated mice on Day 60 p.i. These results suggest that the viral RNA level was too low to be detectable by nested RT-PCR in the CNS on 60 day p.i. Furthermore, only two of the six UR-inoculated mice showed mild inflammatory infiltration, without a demyelinating lesion, in the white matter of the spinal cord and the lateral ventricle.

These results indicated that SAFV-3 was only mildly neurovirulent in young ddY and BALB/c mice when inoculated intracerebrally. Both strains showed low infectivity and neurotropism in the brains of these young mice; however, the immune response of mice infected with the two SAFV-3 strains differed, particularly that in the cerebellum of young BALB/c mice. The observed body weight changes, coupled with the inflammatory infiltrates in the cerebellum, suggest that young ddY and BALB/c mice have different susceptibilities or immune responses to infection by SAFV-3 isolates. Young BALB/c mice (Th-2 prone inbred mice) showed a stronger immune response to SAFV-3 infection than ddY mice.

### SAFV-3 infection via the mucosa in young BALB/c mice

**Fig 10:**
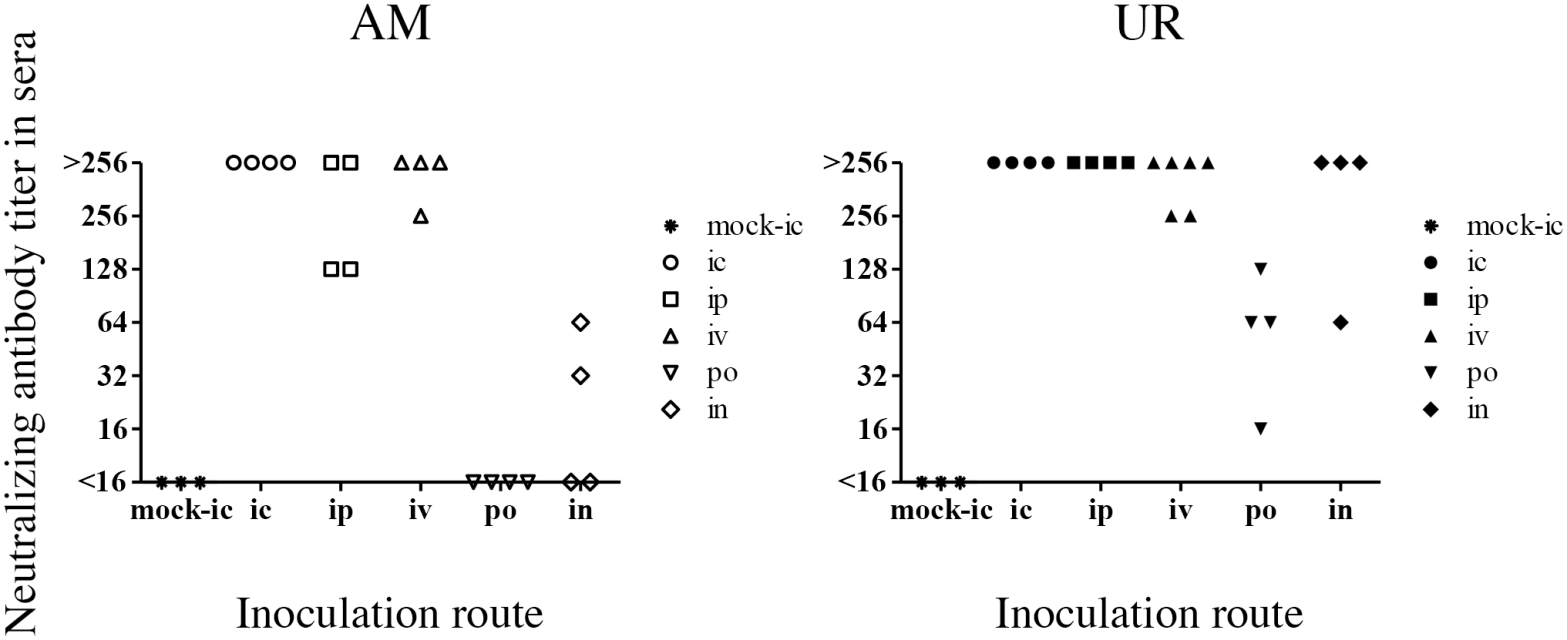
Titers of neutralizing antibodies against SAFV-3 in sera from young BALB/c mice. On Day 21 post-inoculation, sera were obtained from mice inoculated intracerebrally (ic), intraperitoneally (ip), intravenously (iv), orally (po), or intranasally (in) with the aseptic meningitis (AM) or upper respiratory (UR) strains of SAFV-3 (n = 4 or 6 mice per group). All mice inoculated with the UR strain, but not those inoculated with the AM strain, via the oral or intranasal routes, generated neutralizing antibodies.

Finally, we examined the neuroinvasiveness and infectivity of SAFV-3 by inoculating BALB/c mice via different routes. Because the oral cavity of neonatal mice was positive for viral antigens after intracerebral inoculation with the UR strain (Fig 2A), we speculated that SAFV-3 might infect young mice through the mucosa. To explore this hypothesis, we infected young BALB/c mice with the AM and UR strains of SAFV-3 (10^4^ CCID_50_ per mouse) via various routes (intraperitoneal, intravenous, oral, and intranasal). None of the animals showed clinical neurological signs. In addition, no histopathological changes were observed in the CNS (including the olfactory bulb) or other organs on Days 3, 8, and 21 p.i. However, on Day 3 post-intraperitoneal inoculation, immunohistochemical analysis revealed viral antigen-positive cells in the pancreas of all three AM-inoculated mice and one of the three UR-inoculated mice (S8 Fig). Similarly, on Day 3 post-intravenous inoculation of UR, viral antigens were detected in the pancreas in one of three mice. However, no histopathological changes or viral antigens were observed in the major organs after oral or intranasal inoculation with either virus strain. Interestingly, very mild and focal inflammatory infiltrations (in the absence of viral antigen) were seen in the nasal mucosa of mice inoculated with UR via the oral and intranasal routes. No virus was detected in the feces on Days 3 and 8 after oral infection. We then measured the levels of anti-SAFV-neutralizing antibodies in the sera of mice on Day 21 p.i., to examine seroconversion against each inoculated strain. All of the young BALB/c mice seroconverted after intracerebral, intraperitoneal, or intravenous inoculation with SAFV-3 (Fig 10). Notably, oral and intranasal inoculation with the UR strain induced seroconversion in young BALB/c mice. By contrast, mice orally administered the AM strain did not seroconvert. After intranasal administration, two out of four animals showed low levels of neutralizing antibodies against the inoculated virus. These results suggested that the UR strain, but not the AM strain, infected young BALB/c mice through “natural” routes, including the oral and nasal mucosa.

## Discussion

Here, we showed that two clinical SAFV-3 isolates, an AM strain isolated from a patient with aseptic meningitis and a UR strain isolated from a patient with an upper respiratory infection, induced mild neuropathogenesis in neonatal and young mice. Neither strain caused severe neurological manifestations, such as paralysis or fatal encephalitis, in neonatal or young mice. Neonatal ddY mice intracerebrally inoculated with the UR strain showed poor (but not fatal) weight gain, and neonatal ddY mice intracerebrally inoculated with the AM strain showed mild clinical signs, such as tremors; however, these signs were minor and transient. Immunohistochemical analysis revealed that both SAFV-3 isolates infected glial cells, but not large pyramidal neurons, in neonatal mice. However, histopathological investigation revealed that both strains induced an aseptic meningitis (lasting at least 3 weeks) in the brains of neonatal mice after intracerebral or intraperitoneal inoculation; thus both SAFV-3 strains were mildly neuropathogenic, neurotropic, and neurovirulent in neonatal ddY mice. There were some differences between the two SAFV-3 strains in terms of infectivity and cell tropism in the murine neonatal model. For instance, the ependymal cells and mucosal epithelia of the oral cavity and the tooth germ in neonatal mice were infected following intracerebral infection with the UR, but not the AM, strain. In addition, the UR strain infected the mucosa in young mice following intranasal or oral inoculation; thus this animal model recapitulates the potential infectivity of the UR strain observed in human upper respiratory tract infections. Therefore, both the neonatal and young mouse models will be useful for future studies aimed at elucidating the pathogenesis of different SAFV-3 strains.

The present study highlights the similarities and differences between SAFV-3 and other cardioviruses. Theiler's murine encephalomyelitis virus (TMEV) is a common enteric pathogen in mice and causes poliomyelitis-like flaccid paralysis and a chronic progressive white matter-demyelinating disease, which is considered a relevant viral infection model of human multiple sclerosis [51–53]. Neurovirulent strains of TMEV mainly infect the cerebral cortex, hippocampus, and spinal cord after experimental inoculation [54–56]. Virus-mediated neurological disorders are caused by direct lytic infection of neurons and oligodendroglia and by secondary immune-mediated destruction of infected cells [57]. We found that the UR-inoculated neonatal mice intracerebrally showed foci of demyelination with microglia infiltrations in the spinal cord on day 21 p.i. This seems to be an early phase of demyelination after viral infection, which is likely to be due to TMEV infection. However, SAFV-3 infected adult mice did not show any lesion associated with demyelinating disease. Interestingly, the SAFV-3 strains examined herein were neurotropic for glial cells in the cerebellum but not for oligodendroglia; however, no study has reported a similar tropism for TMEV [54]. Additionally, there is no evidence for an association between SAFV and human multiple sclerosis [13, 58]. The pathological process of demyelination may be different between SAFV and TMEV. In the present study, SAFV-3 infected the pancreas of young mice after intraperitoneal inoculation. Another cardiovirus, *Encephalomyocarditis virus*, also infects the pancreas of mice and causes type 1 diabetes [3, 5]; thus, SAFV-3 may have the potential to induce diabetes in mice. Similar to other murine enteric cardioviruses, the UR strain infected young mice via the mucosal route. Therefore, this mucosal infection model may mimic SAFV infection in humans and prove useful for furthering our understanding of the pathogenesis of SAFV. However, the routes of viral infection following oral and intranasal inoculation remain unclear.

The difference between the two SAFV-3 strains in terms of their ability to infect epithelial cells and neurons in the cerebellum may be related to the specific receptors used, or to factors that affect viral replication. Although the AM and UR strains differed by only a single amino acid in the VP2 protein; there were seven amino acid differences in the non-structural proteins (L, 2A, 2C, 3B, 3C, and 3D). The capsid protein of cardioviruses contains four loop structures, loops I and II in VP1, and puffs A and B in VP2, all of which are involved in receptor binding [13, 59, 60]. The leader protein of TMEV plays an important role in viral replication by inhibiting alpha/beta interferon production [61, 62] and inducing apoptosis [63, 64]. It also plays a role in neurovirulence [65]. In the present study, the AM and UR strains differed in terms of virus replication and induction of type 1 IFN expression in infected mice. The dissimilarity in the amino acid sequences of the two SAFV-3 isolates may have contributed to the observed differences in tissue tropism, viral replication, and type 1 IFN expression in the two mouse models. Notably, the expression of type 1 IFN in the cerebellum of AM-inoculated young mice was significantly greater than that in UR-inoculated mice. This discrepancy could be related to differences in cell tropism in these mice.

The AM strain showed greater tropism for the cerebellum than the UR strain. Nielsen *et al*. detected SAFV in the CSF and fecal samples from a 16-month-old child suffering from monosymptomatic ataxia caused by cerebellitis [35]. The neural signs, fluctuating from an insecure gait to walking into things and falling, remitted completely without sequelae during the next 2 months. In the present study, AM-inoculated neonatal mice showed neurological signs such as ataxia, rolling, and elevated tails on Days 7 and 8 p.i.; these mice also recovered rapidly. Interestingly, viral antigens were detected in glial cells in the cerebellum (aka Bergmann glia) but not in neural (Purkinje) cells. Bergmann glia and Purkinje cells, which construct glial microdomains, interact with growing neurites [66]. Cerebellar ataxia is generally thought to be caused by the loss of Purkinje cells. Therefore, we speculate that the SAFV-infected Bergmann glia in the present study affected the function of the Purkinje cells, leading to the cerebellar ataxia observed in neonatal mice.

Highly neurotropic viruses such as poliovirus, enterovirus 71, and neurovirulent flaviviruses, infect the CNS and cause viral meningitis or encephalomyelitis, which is characterized by neuronal damage and perivascular inflammation [67–70]. The preferential target of these neurotropic viruses is the large pyramidal neurons in the human CNS (and in that of animal models) [38, 71, 72]. In this study, the two SAFV-3 strains only infected neural progenitor cells and glial cells around the lateral ventricles and cerebellum in both neonatal and young mice. Neither infected the large pyramidal neurons in the brain cortex, brain stem, spinal cord, and cerebellum. This tropism of SAFV-3 could reflect the neuropathogenesis, such as meningitis and cuffing, observed in the brains of these mice (and possibly that observed in the human brain).

## Conclusions

In summary, we examined the neuropathogenesis of SAFV-3 in two mouse models and found that although SAFV-3 is a candidate neuropathogenic agent, it is different from the highly neurotropic enteroviruses, such as poliovirus, enterovirus 71, and coxsackieviruses A and B. The present *in vivo* comparative study using virological, pathological, and immunological methods to examine the effects of clinical viral isolates has further elucidated the pathogenesis of SAFV. However, future studies on the neuropathogenesis of SAFV will be required for complete understanding of this virus.

## Supporting Information

S1 Fig Inflammatory infiltrate in the brains of neonatal ddY mice after intracerebral inoculation with SAFV-3. Within 24 h of birth, neonatal ddY mice were inoculated intracerebrally with 10^4^ CCID_50_ (cell culture infectious dose) of the aseptic meningitis (AM) or upper respiratory (UR) strains of SAFV-3. Representative histopathological images of inflammatory infiltration in the brains of neonatal mice on Days 3 and 21 post-inoculation (p.i.) are shown (n = 3, 4, or 7 mice per group). Hematoxylin and eosin (H&E) staining. Bars, 50 μm. On Day 3 p.i., swollen vascular endothelial cells (red arrow, inset) and a mild inflammatory infiltrate with microglia (blue arrow, inset) were seen in the cerebrum/brain stem of UR-inoculated mice (black arrows, panels in second row), but not in that of AM-inoculated mice (panels in first row). On Day 21 p.i., perivascular cuffing with mononuclear cell infiltration was observed in the brain stem/cerebellum of UR-inoculated mice (arrows and insets of fourth row) but not in those of AM-inoculated mice (panels in third row). Original magnification, 400×; insets, 1,000×.
(TIF)

S2 Fig Histopathology of the heart in neonatal ddY mice after intracerebral inoculation with SAFV-3. On Day 3 post-inoculation (p.i.), hearts were obtained from neonatal ddY mice after intracerebral inoculation with 10^4^ CCID_50_ (cell culture infectious dose) of the aseptic meningitis (AM) and upper respiratory (UR) strains of SAFV-3. Hematoxylin and eosin (H&E) staining and immunohistochemical analysis with an anti-SAFV-3 antibody (anti-SAFV). Bar, 20 μm. The viral antigen-positive cells were seen in the cardiac muscle cells in both AM- and UR-inoculated mice. CP, Cavity of Pericardium. Original magnification, 1,000×.
(TIF)

S3 Fig Histopathology of neonatal ddY mice after intraperitoneal inoculation with SAFV-3. Within 24 h of birth, neonatal ddY mice were intraperitoneally inoculated with 10^4^ CCID_50_ (cell culture infectious dose) of the aseptic meningitis (AM) and upper respiratory (UR) strains of SAFV-3. Representative histopathological findings of viral infection in neonatal mice on Day 3 post-inoculation (p.i.) (A) and of inflammatory infiltration on Day 21 p.i. (B) are shown. Hematoxylin and eosin staining (H&E) and immunohistochemical analysis with an anti-SAFV-3 antibody (anti-SAFV). Bar, 50 μm. Very slight or mild histopathological changes were observed around the fourth ventricle and in the cerebellum of both AM- and UR-inoculated mice (A). The glial cells of the brain stem and cerebellum and the skeletal muscle cells of abdominal muscle in AM-inoculated mice were virus antigen-positive (brown). By contrast, the ependymal and glial cells of the brain stem and cerebellum, and the skeletal muscle cells and tooth germ cells, of UR-inoculated mice were viral antigen-positive (A). The cytoplasm of degenerated glial cells (with condensation nuclei) was positive for viral antigens (insets show the brain stem and cerebellum). On Day 21 p.i., perivascular cuffing and mononuclear cell infiltration were observed in the brain stem and cerebellum of UR-inoculated mice, but not in those of AM-inoculated mice (B, arrows and insets). Original magnification, 400×; insets, 1,000×.
(TIF)

S4 Fig Identification of SAFV-3-infected cells in neonatal ddY mouse brain after intraperitoneal inoculation. Within 24 h of birth, neonatal ddY mice were inoculated intraperitoneally with 10^4^ CCID_50_ (cell culture infectious dose) of the aseptic meningitis (AM) or upper respiratory (UR) strain of SAFV-3. Double immunofluorescent images showing viral antigens (red) and markers (green) for Musashi-1^+^ neural progenitor cells, GFAP^+^ astrocytes, and GLAST^+^ radial astrocytes in the brains of mice on Day 3 post-inoculation are presented. Musashi-1^+^ neural progenitor cells and GFAP^+^ glial cells around the ventricle of the brain stem and GLAST^+^ glial cells in the cerebellum from both AM- and UR-inoculated mice were also positive for viral antigen. Arrows, viral antigen-positive and neural marker-positive cells. Original magnification, 600×.
(TIF)

S5 Fig Histopathology of young ddY mice after intracerebral inoculation with SAFV-3. On Day 3 post-inoculation (p.i.), brains were obtained from young ddY mice after intracerebral inoculation with 10^4^ CCID_50_ (cell culture infectious dose) of the aseptic meningitis (AM) and upper respiratory (UR) strains of SAFV-3. Hematoxylin and eosin (H&E) staining and immunohistochemical analysis with an anti-SAFV-3 antibody (anti-SAFV). Bar, 50 μm. Nerve cells were degenerated (arrows) with mild inflammatory infiltration (asterisk), and the cerebral medulla was positive for viral antigens (insets, left panels). Several viral antigen-positive cells were seen in lesions in both AM- and UR-inoculated mice. Nerve cells in the cerebral cortex were negative for viral antigen. Viral antigen-positive cells were observed in the molecular layer of the cerebellum of both AM- and UR-inoculated mice (insets, right panels). Purkinje cells were negative for viral antigens. Cx, Cortex; Md, Medulla, LV, Lateral Ventricle. Original magnification, 400×; insets, 1,000×.
(TIF)

S6 Fig Identification of SAFV-3-infected cells in the young ddY mouse brain after intracerebral inoculation. Young ddY mice were inoculated intracerebrally with 10^4^ CCID_50_ (cell culture infectious dose) of the aseptic meningitis (AM) or upper respiratory (UR) strain of SAFV-3. Double immunofluorescent images showing viral antigens (red) and markers (green) for Musashi-1^+^ neural progenitor, GFAP^+^, and GLAST^+^ (astrocytes) cells in the brains of mice on Day 3 post-inoculation are presented. In both of the AM- and UR-inoculated mice, Musashi-1^+^ progenitor neural cells, and GFAP^+^ or GLAST^+^ glial cells within the cerebral medulla were positive for viral antigen. GFAP^+^ glial fibers within the cerebellum were positive for viral antigen (Lower panels). Arrows, viral antigen-positive and neural marker-positive cells. Original magnification, 600×.
(TIF)

S7 Fig Identification of SAFV-3-infected cells in the young BALB/c mouse brain after intracerebral inoculation. Young BALB/c mice were inoculated intracerebrally with 10^4^ CCID_50_ (cell culture infectious dose) of the aseptic meningitis (AM) or upper respiratory (UR) strain of SAFV-3. Double immunofluorescent images showing viral antigens (red) and markers (green) for Musashi-1^+^ neural progenitor cells, and GLAST^+^ and GFAP^+^ astrocytes in the brains of mice on Day 3 post-inoculation are presented. Viral antigen-positive cells were identified as Musashi-1^+^, GFAP^+^, or GLAST^+^ in the cerebral medulla. Arrows, viral antigen-positive and neural marker-positive cells. Original magnification, 600×.
(TIF)

S8 Fig Histopathology of the pancreas in young BALB/c mice after intraperitoneal inoculation with SAFV-3. On Day 3 post-inoculation (p.i.), pancreata were obtained from BALB/c mice after intraperitoneal inoculation with 10^4^ CCID_50_ (cell culture infectious dose) of the aseptic meningitis (AM) and upper respiratory (UR) strains of SAFV-3. Hematoxylin and eosin (H&E) staining and immunohistochemical analysis with an anti-SAFV-3 antibody (anti-SAFV). Bar, 20 μm. Viral antigen-positive cells were seen in acinar cells of the pancreas from both AM- and UR- inoculated mice. The viral antigen-positive cells had a basophilic cytoplasm (arrows). Pancreatic islet cells were negative for viral antigens. Original magnification, 1,000×.
(TIF)

S1 Table Histopathological data of neonatal ddY mice after intraperitoneal inoculation with Saffold virus. (DOCX)

S2 Table Detection of viral genome and viral antigen in the brains of adult BALB/c mice after intracerebral inoculation with Saffold virus. (DOCX)

## References

[1] Jones MS, Lukashov VV, Ganac RD, Schnurr DP. Discovery of a novel human picornavirus in a stool sample from a pediatric patient presenting with fever of unknown origin. J Clin Microbiol. 2007;45(7):2144–50. Epub 2007/04/27. 10.1128/JCM.00174-07 17460053 PMC1933019

[2] Dickinson L, Griffiths AJ. The pathogenesis of experimental infections with encephalomyocarditis (EMC) virus. Br J Exp Pathol. 1966;47(1):35–44. Epub 1966/02/01. 4286251 PMC2094592

[3] Craighead JE, McLane MF. Diabetes mellitus: induction in mice by encephalomyocarditis virus. Science. 1968;162(3856):913–4. Epub 1968/11/22. . 4300932 10.1126/science.162.3856.913

[4] Dal Canto MC, Lipton HL. Multiple sclerosis. Animal model:Theiler's virus infection in mice. Am J Pathol. 1977;88(2):497–500. Epub 1977/08/01. 195474 PMC2032181

[5] Yoon JW, McClintock PR, Onodera T, Notkins AL. Virus-induced diabetes mellitus. XVIII. Inhibition by a nondiabetogenic variant of encephalomyocarditis virus. J Exp Med. 1980;152(4):878–92. Epub 1980/10/01. 6252275 10.1084/jem.152.4.878 PMC2185964

[6] Rodriguez M, Oleszak E, Leibowitz J. Theiler's murine encephalomyelitis: a model of demyelination and persistence of virus. Crit Rev Immunol. 1987;7(4):325–65. Epub 1987/01/01. . 2827957

[7] Hubbard GB, Soike KF, Butler TM, Carey KD, Davis H, Butcher WI, et al An encephalomyocarditis virus epizootic in a baboon colony. Lab Anim Sci. 1992;42(3):233–9. Epub 1992/06/01. . 1320151

[8] Reddacliff LA, Kirkland PD, Hartley WJ, Reece RL. Encephalomyocarditis virus infections in an Australian zoo. J Zoo Wildl Med. 1997;28(2):153–7. Epub 1997/06/01. . 9279403

[9] Oberste MS, Gotuzzo E, Blair P, Nix WA, Ksiazek TG, Comer JA, et al Human febrile illness caused by encephalomyocarditis virus infection, Peru. Emerg Infect Dis. 2009;15(4):640–6. Epub 2009/04/01. 10.3201/eid1504.081428 19331761 PMC2671410

[10] Canelli E, Luppi A, Lavazza A, Lelli D, Sozzi E, Martin AM, et al Encephalomyocarditis virus infection in an Italian zoo. Virol J. 2010;7:64 Epub 2010/03/20. 10.1186/1743-422X-7-64 20298561 PMC2848215

[11] Czechowicz J, Huaman JL, Forshey BM, Morrison AC, Castillo R, Huaman A, et al Prevalence and risk factors for encephalomyocarditis virus infection in Peru. Vector Borne Zoonotic Dis. 2011;11(4):367–74. Epub 2011/03/15. 10.1089/vbz.2010.0029 . 21395427

[12] Masek-Hammerman K, Miller AD, Lin KC, MacKey J, Weissenbock H, Gierbolini L, et al Epizootic myocarditis associated with encephalomyocarditis virus in a group of rhesus macaques (Macaca mulatta). Vet Pathol. 2012;49(2):386–92. Epub 2011/06/10. 10.1177/0300985811409254 . 21653204

[13] Chiu CY, Greninger AL, Kanada K, Kwok T, Fischer KF, Runckel C, et al Identification of cardioviruses related to Theiler's murine encephalomyelitis virus in human infections. Proc Natl Acad Sci U S A. 2008;105(37):14124–9. Epub 2008/09/05. 10.1073/pnas.0805968105 18768820 PMC2528868

[14] Drexler JF, Luna LK, Stocker A, Almeida PS, Ribeiro TC, Petersen N, et al Circulation of 3 lineages of a novel Saffold cardiovirus in humans. Emerg Infect Dis. 2008;14(9):1398–405. Epub 2008/09/02. 10.3201/eid1409.080570 18760006 PMC2603095

[15] Blinkova O, Kapoor A, Victoria J, Jones M, Wolfe N, Naeem A, et al Cardioviruses are genetically diverse and cause common enteric infections in South Asian children. J Virol. 2009;83(9):4631–41. Epub 2009/02/06. 10.1128/JVI.02085-08 19193786 PMC2668475

[16] Ren L, Gonzalez R, Xiao Y, Xu X, Chen L, Vernet G, et al Saffold cardiovirus in children with acute gastroenteritis, Beijing, China. Emerg Infect Dis. 2009;15(9):1509–11. Epub 2009/10/01. 10.3201/eid1509.081531 19788828 PMC2819865

[17] Xu ZQ, Cheng WX, Qi HM, Cui SX, Jin Y, Duan ZJ. New Saffold cardiovirus in children, China. Emerg Infect Dis. 2009;15(6):993–4. Epub 2009/06/16. 10.3201/eid1506.090109 19523321 PMC2727318

[18] Zoll J, Erkens Hulshof S, Lanke K, Verduyn Lunel F, Melchers WJ, Schoondermark-van de Ven E, et al Saffold virus, a human Theiler's-like cardiovirus, is ubiquitous and causes infection early in life. PLoS Pathog. 2009;5(5):e1000416 Epub 2009/05/05. 10.1371/journal.ppat.1000416 19412527 PMC2670511

[19] Chiu CY, Greninger AL, Chen EC, Haggerty TD, Parsonnet J, Delwart E, et al Cultivation and serological characterization of a human Theiler's-like cardiovirus associated with diarrheal disease. J Virol. 2010;84(9):4407–14. Epub 2010/02/19. 10.1128/JVI.02536-09 20164225 PMC2863762

[20] Ren L, Gonzalez R, Xie Z, Xiao Y, Li Y, Liu C, et al Saffold cardioviruses of 3 lineages in children with respiratory tract infections, Beijing, China. Emerg Infect Dis. 2010;16(7):1158–61. Epub 2010/07/01. 10.3201/eid1607.091682 20587195 PMC3321900

[21] Tsukagoshi H, Masuda Y, Mizutani T, Mizuta K, Saitoh M, Morita Y, et al Sequencing and phylogenetic analyses of Saffold cardiovirus (SAFV) genotype 3 isolates from children with upper respiratory infection in Gunma, Japan. Jpn J Infect Dis. 2010;63(5):378–80. Epub 2010/09/23. . 20859011

[22] Chua KB, Voon K, Yu M, Ali WN, Kasri AR, Wang LF. Saffold virus infection in children, Malaysia, 2009. Emerg Infect Dis. 2011;17(8):1562–4. Epub 2011/08/02. 10.3201/eid1708.101380 21801653 PMC3381576

[23] Dai XQ, Yuan CL, Yu Y, Zhao W, Yang ZB, Cui L, et al Molecular detection of Saffold Virus in children in Shanghai, China. J Clin Virol. 2011;50(2):186–7. Epub 2010/12/17. 10.1016/j.jcv.2010.11.004 . 21159549

[24] Galama J, Lanke K, Zoll J, Roivainen M, van Kuppeveld F. Seroepidemiology of Saffold cardiovirus type 2. Emerg Infect Dis. 2011;17(8):1572–3. Epub 2011/08/02. 10.3201/eid1708.101953 21801659 PMC3381534

[25] Itagaki T, Abiko C, Aoki Y, Ikeda T, Mizuta K, Noda M, et al Saffold cardiovirus infection in children associated with respiratory disease and its similarity to coxsackievirus infection. Pediatr Infect Dis J. 2011;30(8):680–3. Epub 2011/03/10. 10.1097/INF.0b013e31821608a8 . 21386746

[26] Kobayashi M, Tsukagoshi H, Ishioka T, Mizuta K, Noda M, Morita Y, et al Seroepidemiology of Saffold cardiovirus (SAFV) genotype 3 in Japan. J Infect. 2013;66(2):191–3. Epub 2012/10/31. 10.1016/j.jinf.2012.10.022 . 23107837

[27] Naeem A, Hosomi T, Nishimura Y, Alam MM, Oka T, Zaidi SS, et al Genetic diversity of circulating Saffold viruses in Pakistan and Afghanistan. J Gen Virol. 2014;95(Pt 9):1945–57. Epub 2014/06/06. 10.1099/vir.0.066498-0 . 24899154

[28] Khamrin P, Chaimongkol N, Nantachit N, Okitsu S, Ushijima H, Maneekarn N. Saffold cardioviruses in children with diarrhea, Thailand. Emerg Infect Dis. 2011;17(6):1150–2. Epub 2011/07/14. 21749802 10.3201/eid1706.101983 PMC3358219

[29] Branas P, Garcia M, Prieto C, Folgueira L. Saffold virus respiratory infection in children and immunocompromised patients in Spain. J Infect. 2014 Epub 2014/12/03. 10.1016/j.jinf.2014.11.006 . 25459664

[30] Zhang XA, Lu QB, Wo Y, Zhao J, Huang DD, Guo CT, et al Prevalence and genetic characteristics of Saffold cardiovirus in China from 2009 to 2012. Sci Rep. 2015;5:7704 Epub 2015/01/13. 10.1038/srep07704 . 25572936 PMC5378990

[31] Khamrin P, Thongprachum A, Kikuta H, Yamamoto A, Nishimura S, Sugita K, et al Three clusters of Saffold viruses circulating in children with diarrhea in Japan. Infect Genet Evol. 2013;13:339–43. Epub 2012/11/28. 10.1016/j.meegid.2012.11.004 . 23183311

[32] Yodmeeklin A, Khamrin P, Chuchaona W, Saikruang W, Malasao R, Chaimongkol N, et al SAffold viruses in pediatric patients with diarrhea in Thailand. J Med Virol. 2015 Epub 2015/01/15. 10.1002/jmv.24114 . 25583432

[33] Nix WA, Khetsuriani N, Penaranda S, Maher K, Venczel L, Cselko Z, et al Diversity of picornaviruses in rural Bolivia. J Gen Virol. 2013;94(Pt 9):2017–28. Epub 2013/06/28. 10.1099/vir.0.053827-0 . 23804569 PMC4637931

[34] Victoria JG, Kapoor A, Li L, Blinkova O, Slikas B, Wang C, et al Metagenomic analyses of viruses in stool samples from children with acute flaccid paralysis. J Virol. 2009;83(9):4642–51. Epub 2009/02/13. 10.1128/JVI.02301-08 19211756 PMC2668503

[35] Nielsen AC, Bottiger B, Banner J, Hoffmann T, Nielsen LP. Serious invasive Saffold virus infections in children, 2009. Emerg Infect Dis. 2012;18(1):7–12. Epub 2012/01/21. 10.3201/eid1801.110725 22261113 PMC3310106

[36] Himeda T, Hosomi T, Asif N, Shimizu H, Okuwa T, Muraki Y, et al The preparation of an infectious full-length cDNA clone of Saffold virus. Virol J. 2011;8:110 Epub 2011/03/10. 10.1186/1743-422X-8-110 21385468 PMC3062622

[37] Pallansch MA, Oberste MS, Whitton JL. Enteroviruses: Polioviruses, coxsackieviruses, echoviruses, and newer enteroviruses In: Knipe DM, Howley PM, editors. Fields Virology. One. 6th ed Philadelphia: Wolters Kluwer, Lippincott Williams & Wilkins; 2013 p. 490–530.

[38] Ong KC, Badmanathan M, Devi S, Leong KL, Cardosa MJ, Wong KT. Pathologic characterization of a murine model of human enterovirus 71 encephalomyelitis. J Neuropathol Exp Neurol. 2008;67(6):532–42. Epub 2008/06/04. 10.1097/NEN.0b013e31817713e7 . 18520772

[39] Feuer R, Mena I, Pagarigan RR, Harkins S, Hassett DE, Whitton JL. Coxsackievirus B3 and the neonatal CNS: the roles of stem cells, developing neurons, and apoptosis in infection, viral dissemination, and disease. Am J Pathol. 2003;163(4):1379–93. Epub 2003/09/26. 10.1016/S0002-9440(10)63496-7 14507646 PMC1868316

[40] Chen YC, Yu CK, Wang YF, Liu CC, Su IJ, Lei HY. A murine oral enterovirus 71 infection model with central nervous system involvement. J Gen Virol. 2004;85(Pt 1):69–77. Epub 2004/01/14. . 14718621 10.1099/vir.0.19423-0

[41] Khong WX, Yan B, Yeo H, Tan EL, Lee JJ, Ng JK, et al A non-mouse-adapted enterovirus 71 (EV71) strain exhibits neurotropism, causing neurological manifestations in a novel mouse model of EV71 infection. J Virol. 2012;86(4):2121–31. Epub 2011/12/02. 10.1128/JVI.06103-11 22130542 PMC3302383

[42] Wang L, Dong C, Chen DE, Song Z. Coxsackievirus-induced acute neonatal central nervous system disease model. Int J Clin Exp Pathol. 2014;7(3):858–69. Epub 2014/04/04. 24696707 PMC3971288

[43] Hertzler S, Liang Z, Treso B, Lipton HL. Adaptation of Saffold virus 2 for high-titer growth in mammalian cells. J Virol. 2011;85(14):7411–8. Epub 2011/05/06. 10.1128/JVI.00265-11 21543476 PMC3126574

[44] Li TC, Ami Y, Suzaki Y, Yasuda SP, Yoshimatsu K, Arikawa J, et al Characterization of full genome of rat hepatitis E virus strain from Vietnam. Emerg Infect Dis. 2013;19(1):115–8. Epub 2012/12/25. 10.3201/eid1901.121007 23260149 PMC3558001

[45] Yamazaki T, Kishimoto K, Ezaki O. The ddY mouse: a model of postprandial hypertriglyceridemia in response to dietary fat. J Lipid Res. 2012;53(10):2024–37. Epub 2012/06/28. 10.1194/jlr.M023713 22735545 PMC3435536

[46] Iwata-Yoshikawa N, Uda A, Suzuki T, Tsunetsugu-Yokota Y, Sato Y, Morikawa S, et al Effects of Toll-Like Receptor Stimulation on Eosinophilic Infiltration in Lungs of BALB/c Mice Immunized with UV-Inactivated Severe Acute Respiratory Syndrome-Related Coronavirus Vaccine. J Virol. 2014;88(15):8597–614. Epub 2014/05/23. 10.1128/JVI.00983-14 . 24850731 PMC4135953

[47] Kuribayashi S, Sakoda Y, Kawasaki T, Tanaka T, Yamamoto N, Okamatsu M, et al Excessive cytokine response to rapid proliferation of highly pathogenic avian influenza viruses leads to fatal systemic capillary leakage in chickens. PLoS One. 2013;8(7):e68375 Epub 2013/07/23. 10.1371/journal.pone.0068375 23874602 PMC3706397

[48] Ichinohe T, Watanabe I, Ito S, Fujii H, Moriyama M, Tamura S, et al Synthetic double-stranded RNA poly(I:C) combined with mucosal vaccine protects against influenza virus infection. J Virol. 2005;79(5):2910–9. Epub 2005/02/15. 10.1128/JVI.79.5.2910-2919.2005 15709010 PMC548446

[49] Katano H, Kano M, Nakamura T, Kanno T, Asanuma H, Sata T. A novel real-time PCR system for simultaneous detection of human viruses in clinical samples from patients with uncertain diagnoses. J Med Virol. 2011;83(2):322–30. Epub 2010/12/25. 10.1002/jmv.21962 . 21181930 PMC7166515

[50] Onishi T, Ooshima T, Sobue S, Tabata MJ, Kurisu K, Wakisaka S. Calbindin D28k-like immunoreactivity during the formation of the enamel-free area in the rat molar teeth. Anat Rec. 2000;258(4):384–90. Epub 2000/03/29. . 10737856 10.1002/(SICI)1097-0185(20000401)258:4<384::AID-AR6>3.0.CO;2-U

[51] Theiler M. Spontaneous Encephalomyelitis of Mice, a New Virus Disease. J Exp Med. 1937;65(5):705–19. Epub 1937/04/30. 19870629 10.1084/jem.65.5.705 PMC2133518

[52] Dal Canto MC, Kim BS, Miller SD, Melvold RW. Theiler's Murine Encephalomyelitis Virus (TMEV)-Induced Demyelination: A Model for Human Multiple Sclerosis. Methods. 1996;10(3):453–61. Epub 1996/12/01. . 8954856 10.1006/meth.1996.0123

[53] Kim BS, Lyman MA, Kang BS, Kang HK, Lee HG, Mohindru M, et al Pathogenesis of virus-induced immune-mediated demyelination. Immunol Res. 2001;24(2):121–30. Epub 2001/10/12. 10.1385/IR:24:2:121 . 11594451 PMC7091353

[54] Rodriguez M, Roos RP. Pathogenesis of early and late disease in mice infected with Theiler's virus, using intratypic recombinant GDVII/DA viruses. J Virol. 1992;66(1):217–25. Epub 1992/01/01. 1727485 10.1128/jvi.66.1.217-225.1992 PMC238278

[55] Villarreal D, Young CR, Storts R, Ting JW, Welsh CJ. A comparison of the neurotropism of Theiler's virus and poliovirus in CBA mice. Microb Pathog. 2006;41(4–5):149–56. Epub 2006/08/29. 10.1016/j.micpath.2006.01.009 . 16935465

[56] Buckwalter MR, Nga PT, Gouilh MA, Fiette L, Bureau JF, Laird ME, et al Identification of a novel neuropathogenic Theiler's murine encephalomyelitis virus. J Virol. 2011;85(14):6893–905. Epub 2011/05/06. 10.1128/JVI.00274-11 21543488 PMC3126553

[57] Rodriguez M, Leibowitz JL, Lampert PW. Persistent infection of oligodendrocytes in Theiler's virus-induced encephalomyelitis. Ann Neurol. 1983;13(4):426–33. Epub 1983/04/01. 10.1002/ana.410130409 . 6340596

[58] Galama JM, Zoll JG, Lanke KH, de Jong AS, Melief J, Huitinga I, et al Saffold cardiovirus and multiple sclerosis: no evidence for an association. Ann Clin Transl Neurol. 2014;1(8):618–21. Epub 2014/10/31. 10.1002/acn3.82 25356431 PMC4184563

[59] Jnaoui K, Michiels T. Adaptation of Theiler's virus to L929 cells: mutations in the putative receptor binding site on the capsid map to neutralization sites and modulate viral persistence. Virology. 1998;244(2):397–404. Epub 1998/05/28. 10.1006/viro.1998.9134 . 9601508

[60] Zhou L, Luo Y, Wu Y, Tsao J, Luo M. Sialylation of the host receptor may modulate entry of demyelinating persistent Theiler's virus. J Virol. 2000;74(3):1477–85. Epub 2000/01/11. 10627559 10.1128/jvi.74.3.1477-1485.2000 PMC111483

[61] van Pesch V, van Eyll O, Michiels T. The leader protein of Theiler's virus inhibits immediate-early alpha/beta interferon production. J Virol. 2001;75(17):7811–7. Epub 2001/08/03. 11483724 10.1128/JVI.75.17.7811-7817.2001 PMC115023

[62] Delhaye S, van Pesch V, Michiels T. The leader protein of Theiler's virus interferes with nucleocytoplasmic trafficking of cellular proteins. J Virol. 2004;78(8):4357–62. Epub 2004/03/30. 15047849 10.1128/JVI.78.8.4357-4362.2004 PMC374251

[63] Okuwa T, Taniura N, Saito M, Himeda T, Ohara Y. Opposite effects of two nonstructural proteins of Theiler's murine encephalomyelitis virus regulates apoptotic cell death in BHK-21 cells. Microbiol Immunol. 2010;54(10):639–43. Epub 2010/12/15. 10.1111/j.1348-0421.2010.00260.x . 21140599

[64] Stavrou S, Ghadge G, Roos RP. Apoptotic and antiapoptotic activity of L protein of Theiler's murine encephalomyelitis virus. J Virol. 2011;85(14):7177–85. Epub 2011/05/13. 10.1128/JVI.00009-11 21561911 PMC3126581

[65] Calenoff MA, Faaberg KS, Lipton HL. Genomic regions of neurovirulence and attenuation in Theiler murine encephalomyelitis virus. Proc Natl Acad Sci U S A. 1990;87(3):978–82. Epub 1990/02/01. 2153981 10.1073/pnas.87.3.978 PMC53393

[66] Grosche J, Matyash V, Moller T, Verkhratsky A, Reichenbach A, Kettenmann H. Microdomains for neuron-glia interaction: parallel fiber signaling to Bergmann glial cells. Nat Neurosci. 1999;2(2):139–43. Epub 1999/04/09. 10.1038/5692 . 10195197

[67] Agamanolis DP, Leslie MJ, Caveny EA, Guarner J, Shieh WJ, Zaki SR. Neuropathological findings in West Nile virus encephalitis: a case report. Ann Neurol. 2003;54(4):547–51. Epub 2003/10/02. 10.1002/ana.10731 . 14520673

[68] Cushing MM, Brat DJ, Mosunjac MI, Hennigar RA, Jernigan DB, Lanciotti R, et al Fatal West Nile virus encephalitis in a renal transplant recipient. Am J Clin Pathol. 2004;121(1):26–31. Epub 2004/01/31. 10.1309/G23C-P54D-AR1B-CY8L . 14750237

[69] Gelpi E, Preusser M, Garzuly F, Holzmann H, Heinz FX, Budka H. Visualization of Central European tick-borne encephalitis infection in fatal human cases. J Neuropathol Exp Neurol. 2005;64(6):506–12. Epub 2005/06/28. . 15977642 10.1093/jnen/64.6.506

[70] Wong KT, Ng KY, Ong KC, Ng WF, Shankar SK, Mahadevan A, et al Enterovirus 71 encephalomyelitis and Japanese encephalitis can be distinguished by topographic distribution of inflammation and specific intraneuronal detection of viral antigen and RNA. Neuropathol Appl Neurobiol. 2012;38(5):443–53. Epub 2012/01/13. 10.1111/j.1365-2990.2011.01247.x . 22236252

[71] Nagata N, Iwasaki T, Ami Y, Tano Y, Harashima A, Suzaki Y, et al Differential localization of neurons susceptible to enterovirus 71 and poliovirus type 1 in the central nervous system of cynomolgus monkeys after intravenous inoculation. J Gen Virol. 2004;85(Pt 10):2981–9. Epub 2004/09/28. 10.1099/vir.0.79883-0 . 15448361

[72] Kotani O, Iwata-Yoshikawa N, Suzuki T, Sato Y, Nakajima N, Koike S, et al Establishment of a panel of in-house polyclonal antibodies for the diagnosis of enterovirus infections. Neuropathology. 2015;35(2):107–21. Epub 2014/09/28. 10.1111/neup.12171 . 25263613 PMC7168124

