# Supplementary figures and images for: Neuropathogenicity of Two Saffold Virus Type 3 Isolates in Mouse Models
Source: PLoS One. 2016 Feb 1;11(2):e0148184. doi: 10.1371/journal.pone.0148184 (PMC4734772; doi:10.1371/journal.pone.0148184)

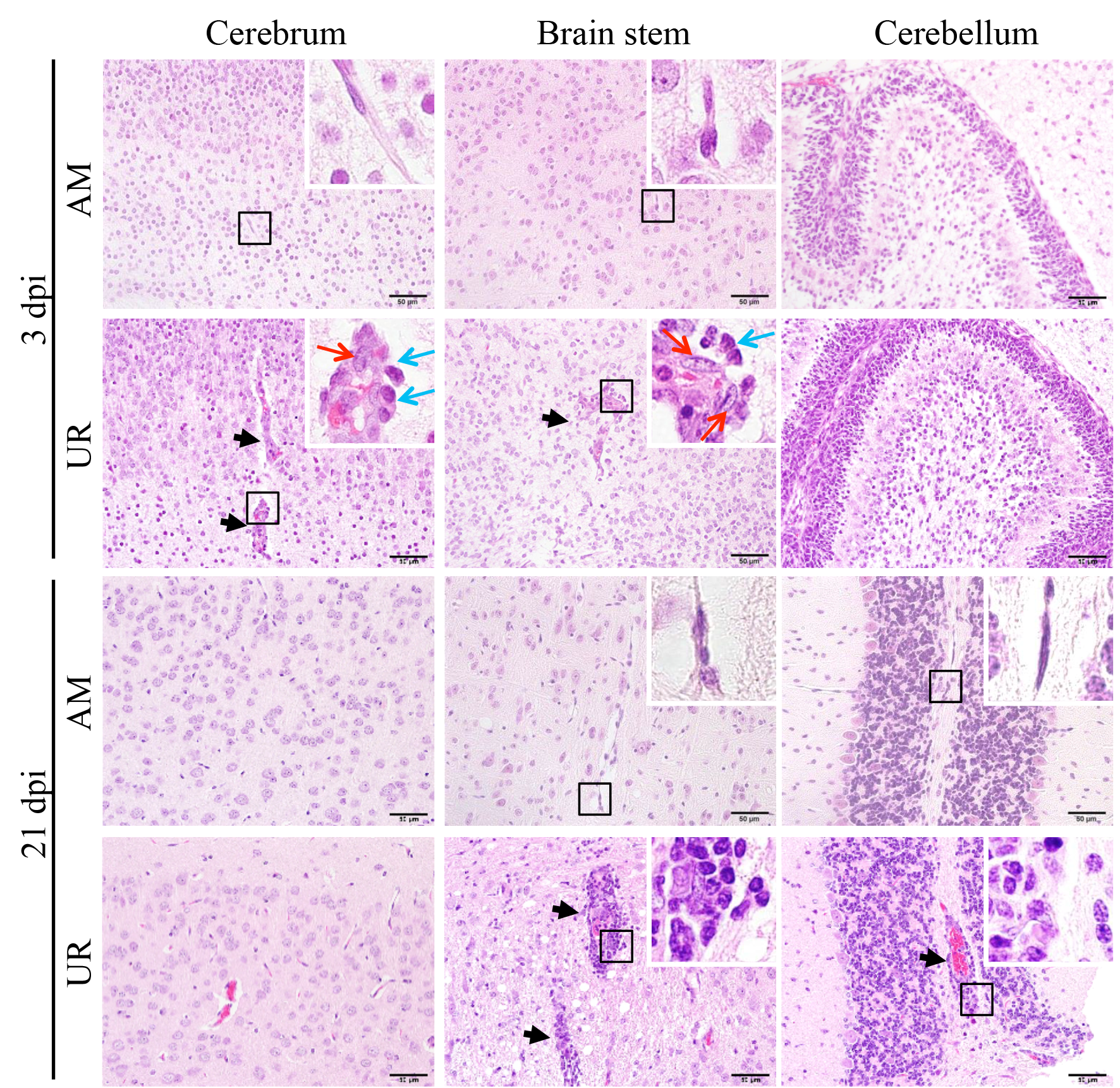

Supplement: S1 Fig — Within 24 h of birth, neonatal ddY mice were inoculated intracerebrally with 104 CCID50 (cell culture infectious dose) of the aseptic meningitis (AM) or upper respiratory (UR) strains of SAFV-3. Representative histopathological images of inflammatory infiltration in the brains of neonatal mice on Days 3 and 21 post-inoculation (p.i.) are shown (n = 3, 4, or 7 mice per group). Hematoxylin and eosin (H&E) staining. Bars, 50 μm. On Day 3 p.i., swollen vascular endothelial cells (red arrow, inset) and a mild inflammatory infiltrate with microglia (blue arrow, inset) were seen in the cerebrum/brain stem of UR-inoculated mice (black arrows, panels in second row), but not in that of AM-inoculated mice (panels in first row). On Day 21 p.i., perivascular cuffing with mononuclear cell infiltration was observed in the brain stem/cerebellum of UR-inoculated mice (arrows and insets of fourth row) but not in those of AM-inoculated mice (panels in third row). Original magnification, 400×; insets, 1,000×. (TIF) [file pone.0148184.s001.tif]

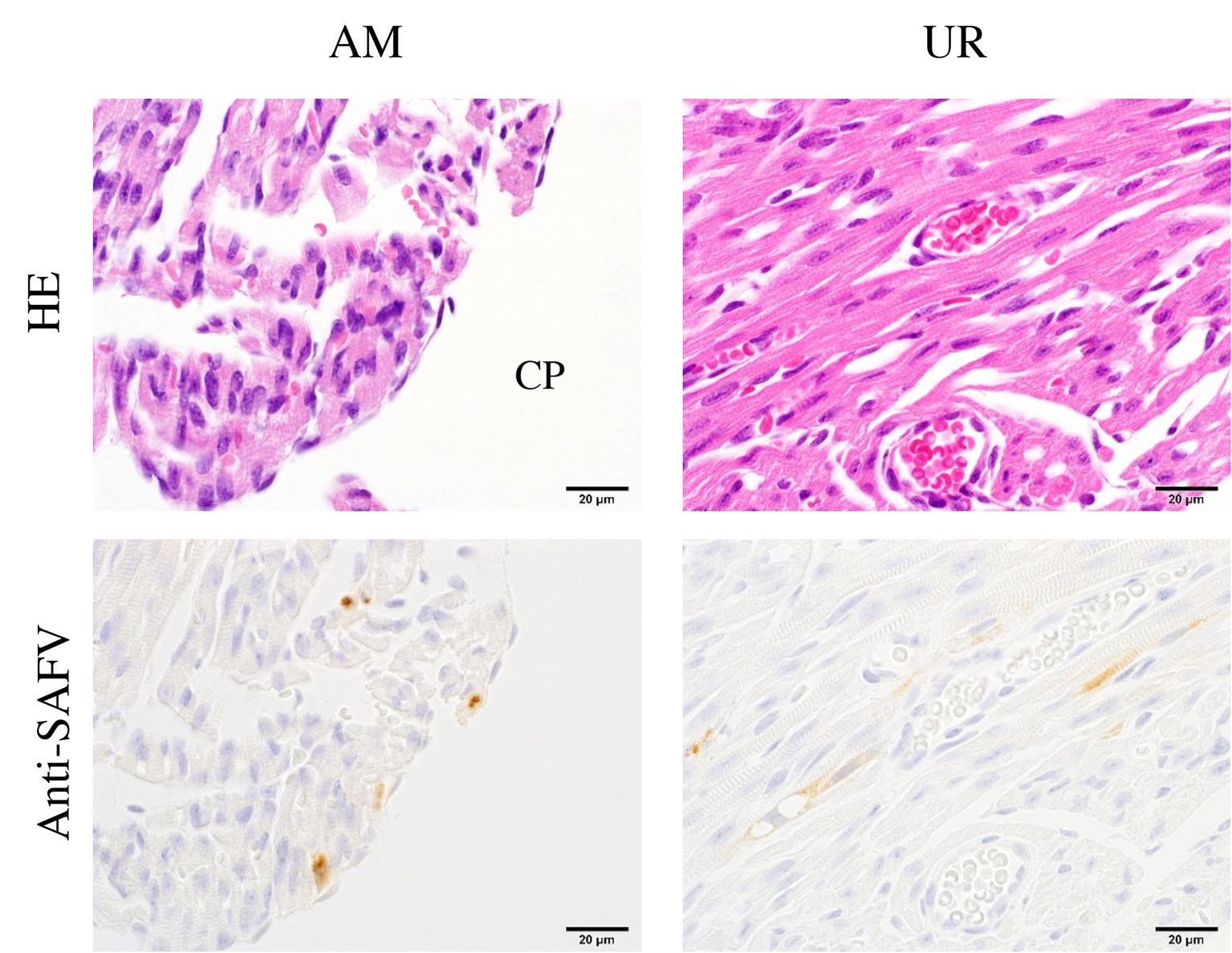

Supplement: S2 Fig — On Day 3 post-inoculation (p.i.), hearts were obtained from neonatal ddY mice after intracerebral inoculation with 104 CCID50 (cell culture infectious dose) of the aseptic meningitis (AM) and upper respiratory (UR) strains of SAFV-3. Hematoxylin and eosin (H&E) staining and immunohistochemical analysis with an anti-SAFV-3 antibody (anti-SAFV). Bar, 20 μm. The viral antigen-positive cells were seen in the cardiac muscle cells in both AM- and UR-inoculated mice. CP, Cavity of Pericardium. Original magnification, 1,000×. (TIF) [file pone.0148184.s002.tif]

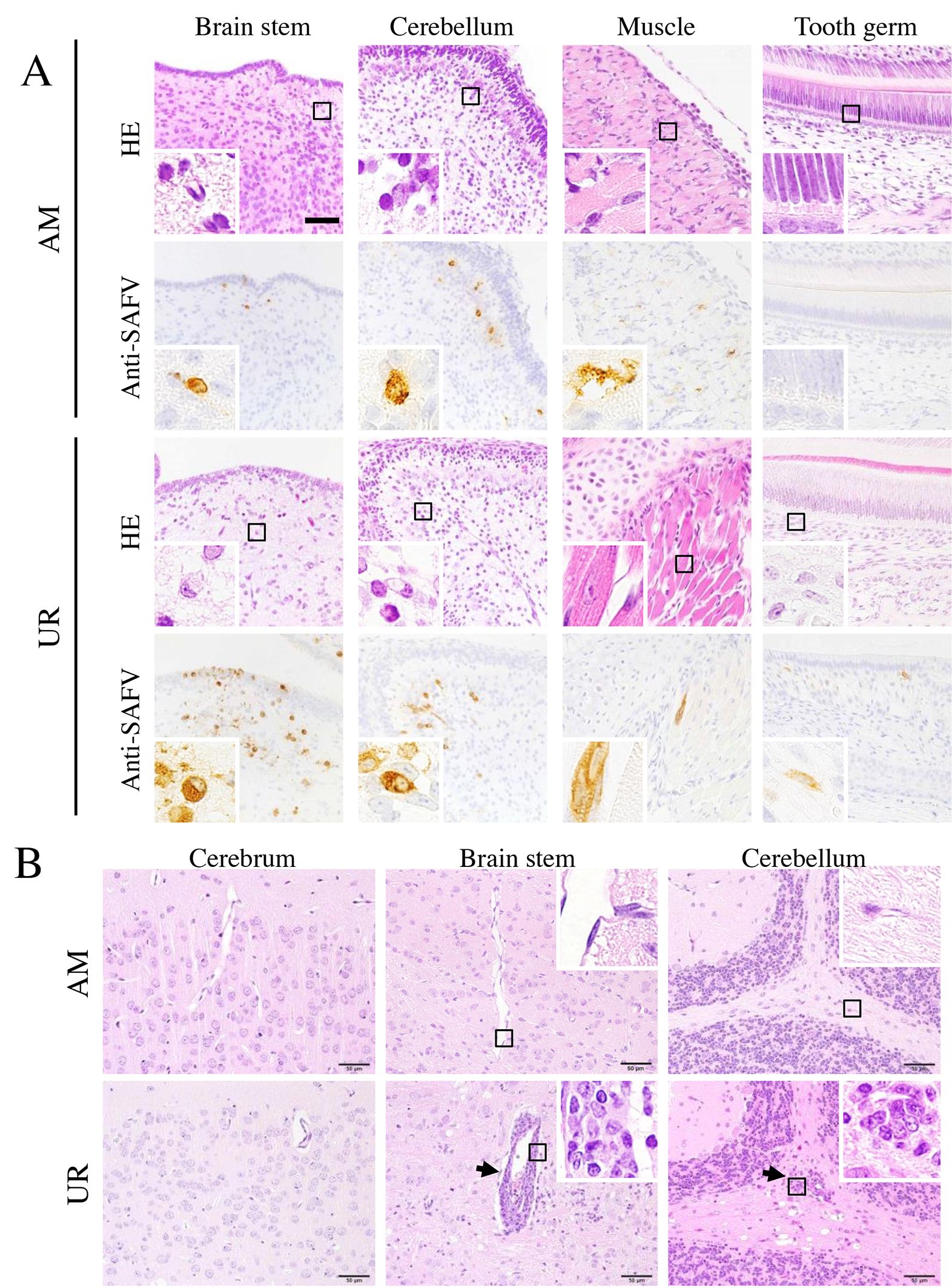

Supplement: S3 Fig — Within 24 h of birth, neonatal ddY mice were intraperitoneally inoculated with 104 CCID50 (cell culture infectious dose) of the aseptic meningitis (AM) and upper respiratory (UR) strains of SAFV-3. Representative histopathological findings of viral infection in neonatal mice on Day 3 post-inoculation (p.i.) (A) and of inflammatory infiltration on Day 21 p.i. (B) are shown. Hematoxylin and eosin staining (H&E) and immunohistochemical analysis with an anti-SAFV-3 antibody (anti-SAFV). Bar, 50 μm. Very slight or mild histopathological changes were observed around the fourth ventricle and in the cerebellum of both AM- and UR-inoculated mice (A). The glial cells of the brain stem and cerebellum and the skeletal muscle cells of abdominal muscle in AM-inoculated mice were virus antigen-positive (brown). By contrast, the ependymal and glial cells of the brain stem and cerebellum, and the skeletal muscle cells and tooth germ cells, of UR-inoculated mice were viral antigen-positive (A). The cytoplasm of degenerated glial cells (with condensation nuclei) was positive for viral antigens (insets show the brain stem and cerebellum). On Day 21 p.i., perivascular cuffing and mononuclear cell infiltration were observed in the brain stem and cerebellum of UR-inoculated mice, but not in those of AM-inoculated mice (B, arrows and insets). Original magnification, 400×; insets, 1,000×. (TIF) [file pone.0148184.s003.tif]

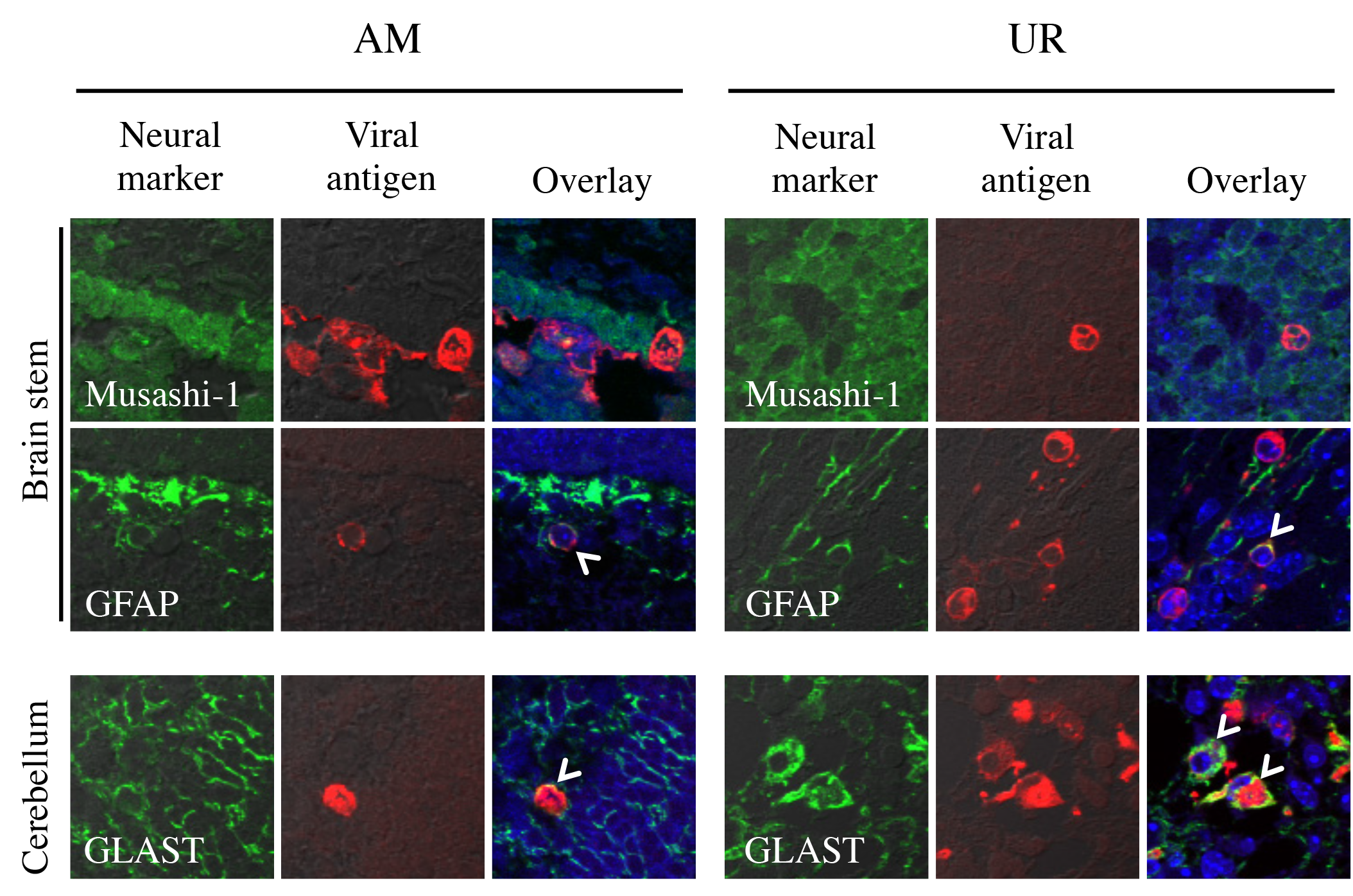

Supplement: S4 Fig — Within 24 h of birth, neonatal ddY mice were inoculated intraperitoneally with 104 CCID50 (cell culture infectious dose) of the aseptic meningitis (AM) or upper respiratory (UR) strain of SAFV-3. Double immunofluorescent images showing viral antigens (red) and markers (green) for Musashi-1+ neural progenitor cells, GFAP+ astrocytes, and GLAST+ radial astrocytes in the brains of mice on Day 3 post-inoculation are presented. Musashi-1+ neural progenitor cells and GFAP+ glial cells around the ventricle of the brain stem and GLAST+ glial cells in the cerebellum from both AM- and UR-inoculated mice were also positive for viral antigen. Arrows, viral antigen-positive and neural marker-positive cells. Original magnification, 600×. (TIF) [file pone.0148184.s004.tif]

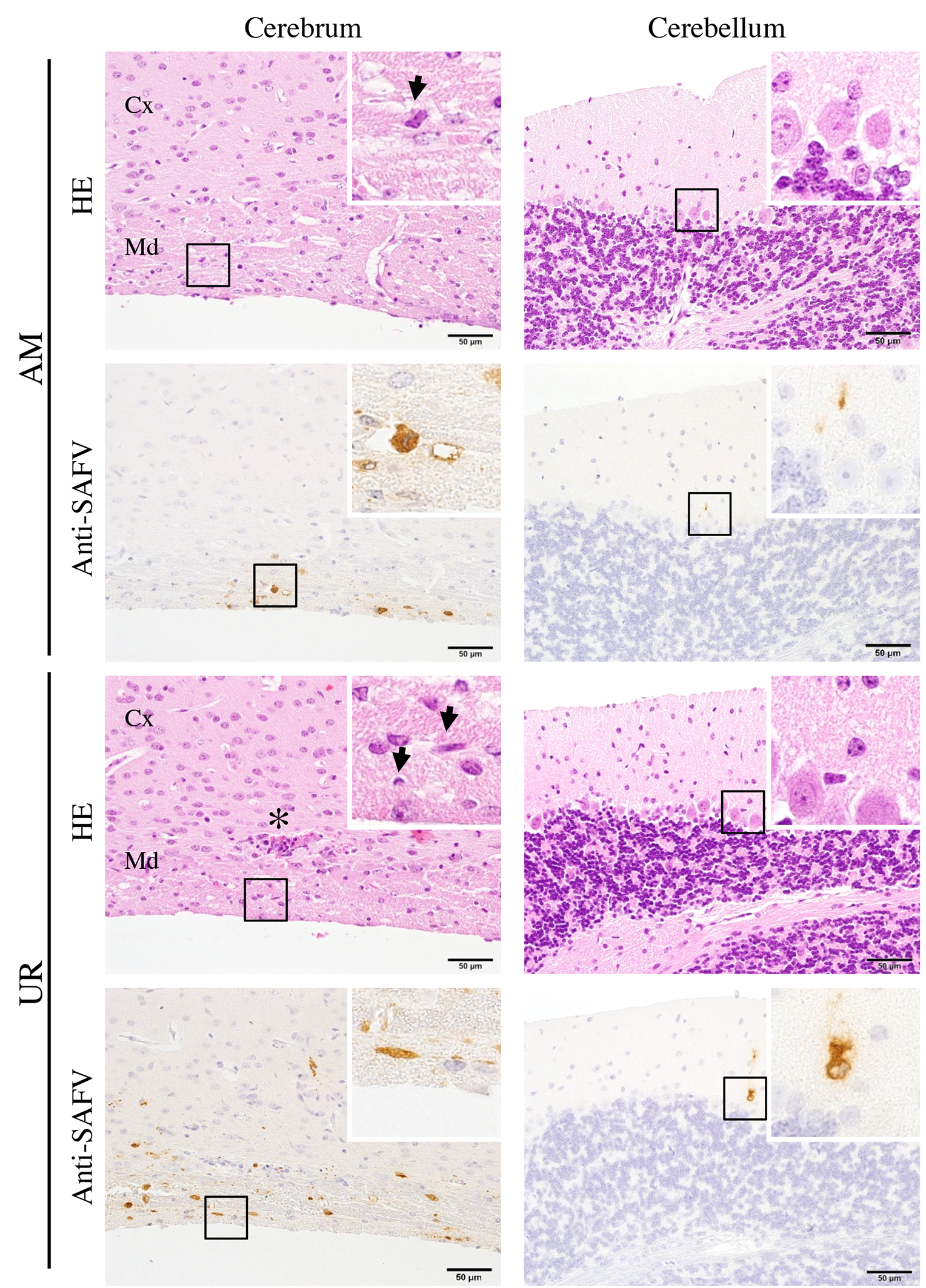

Supplement: S5 Fig — On Day 3 post-inoculation (p.i.), brains were obtained from young ddY mice after intracerebral inoculation with 104 CCID50 (cell culture infectious dose) of the aseptic meningitis (AM) and upper respiratory (UR) strains of SAFV-3. Hematoxylin and eosin (H&E) staining and immunohistochemical analysis with an anti-SAFV-3 antibody (anti-SAFV). Bar, 50 μm. Nerve cells were degenerated (arrows) with mild inflammatory infiltration (asterisk), and the cerebral medulla was positive for viral antigens (insets, left panels). Several viral antigen-positive cells were seen in lesions in both AM- and UR-inoculated mice. Nerve cells in the cerebral cortex were negative for viral antigen. Viral antigen-positive cells were observed in the molecular layer of the cerebellum of both AM- and UR-inoculated mice (insets, right panels). Purkinje cells were negative for viral antigens. Cx, Cortex; Md, Medulla, LV, Lateral Ventricle. Original magnification, 400×; insets, 1,000×. (TIF) [file pone.0148184.s005.tif]

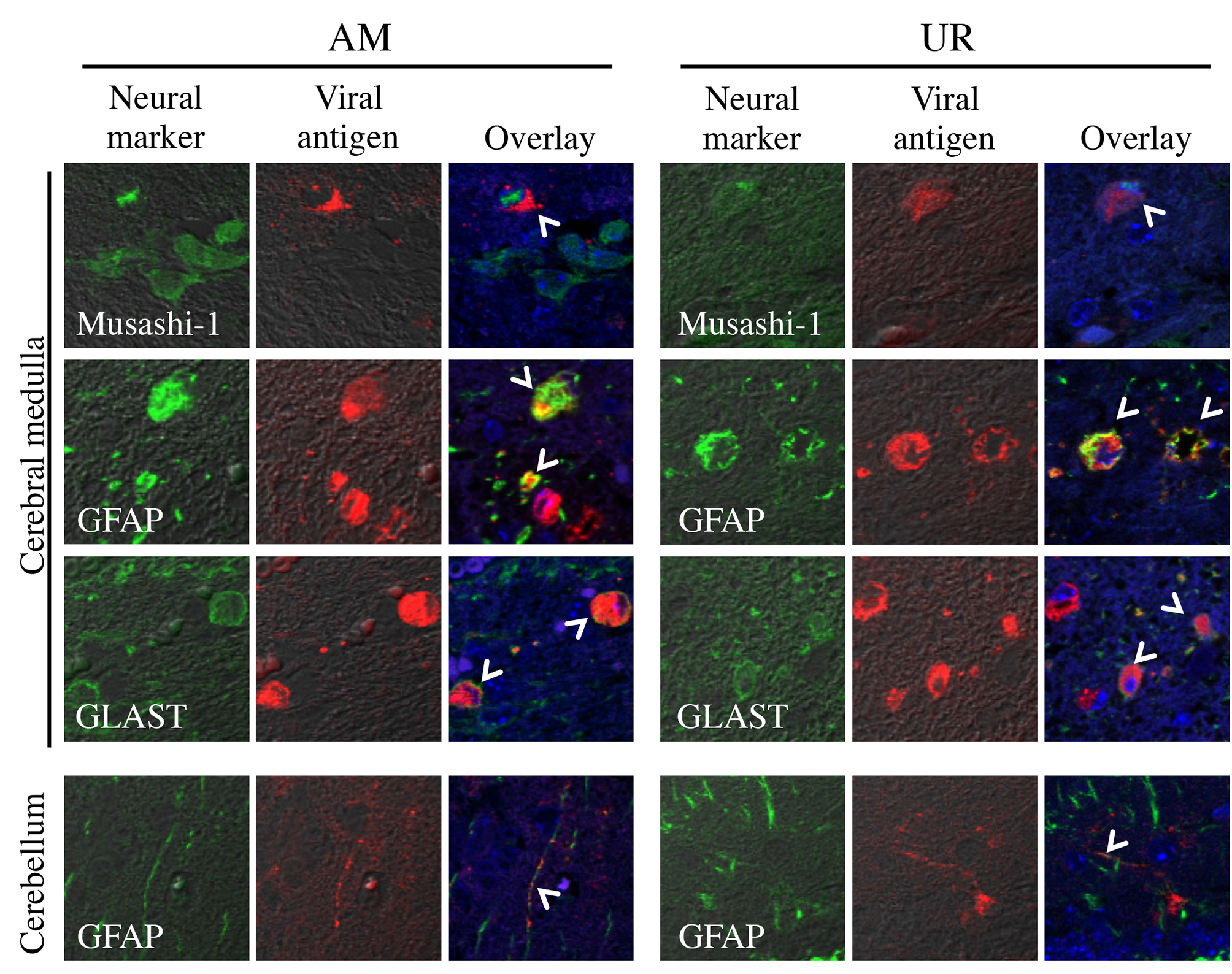

Supplement: S6 Fig — Young ddY mice were inoculated intracerebrally with 104 CCID50 (cell culture infectious dose) of the aseptic meningitis (AM) or upper respiratory (UR) strain of SAFV-3. Double immunofluorescent images showing viral antigens (red) and markers (green) for Musashi-1+ neural progenitor, GFAP+, and GLAST+ (astrocytes) cells in the brains of mice on Day 3 post-inoculation are presented. In both of the AM- and UR-inoculated mice, Musashi-1+ progenitor neural cells, and GFAP+ or GLAST+ glial cells within the cerebral medulla were positive for viral antigen. GFAP+ glial fibers within the cerebellum were positive for viral antigen (Lower panels). Arrows, viral antigen-positive and neural marker-positive cells. Original magnification, 600×. (TIF) [file pone.0148184.s006.tif]

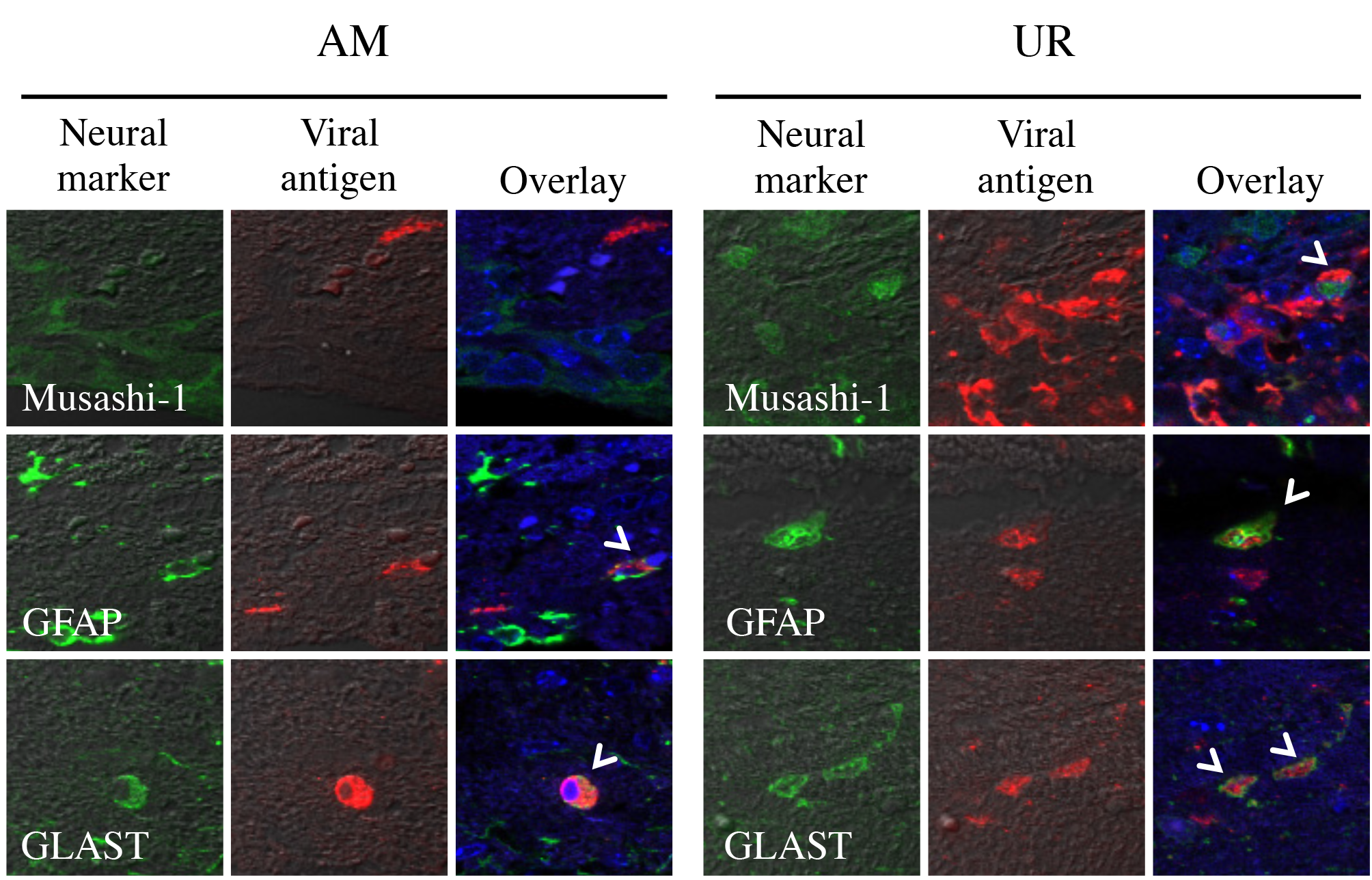

Supplement: S7 Fig — Young BALB/c mice were inoculated intracerebrally with 104 CCID50 (cell culture infectious dose) of the aseptic meningitis (AM) or upper respiratory (UR) strain of SAFV-3. Double immunofluorescent images showing viral antigens (red) and markers (green) for Musashi-1+ neural progenitor cells, and GLAST+ and GFAP+ astrocytes in the brains of mice on Day 3 post-inoculation are presented. Viral antigen-positive cells were identified as Musashi-1+, GFAP+, or GLAST+ in the cerebral medulla. Arrows, viral antigen-positive and neural marker-positive cells. Original magnification, 600×. (TIF) [file pone.0148184.s007.tif]

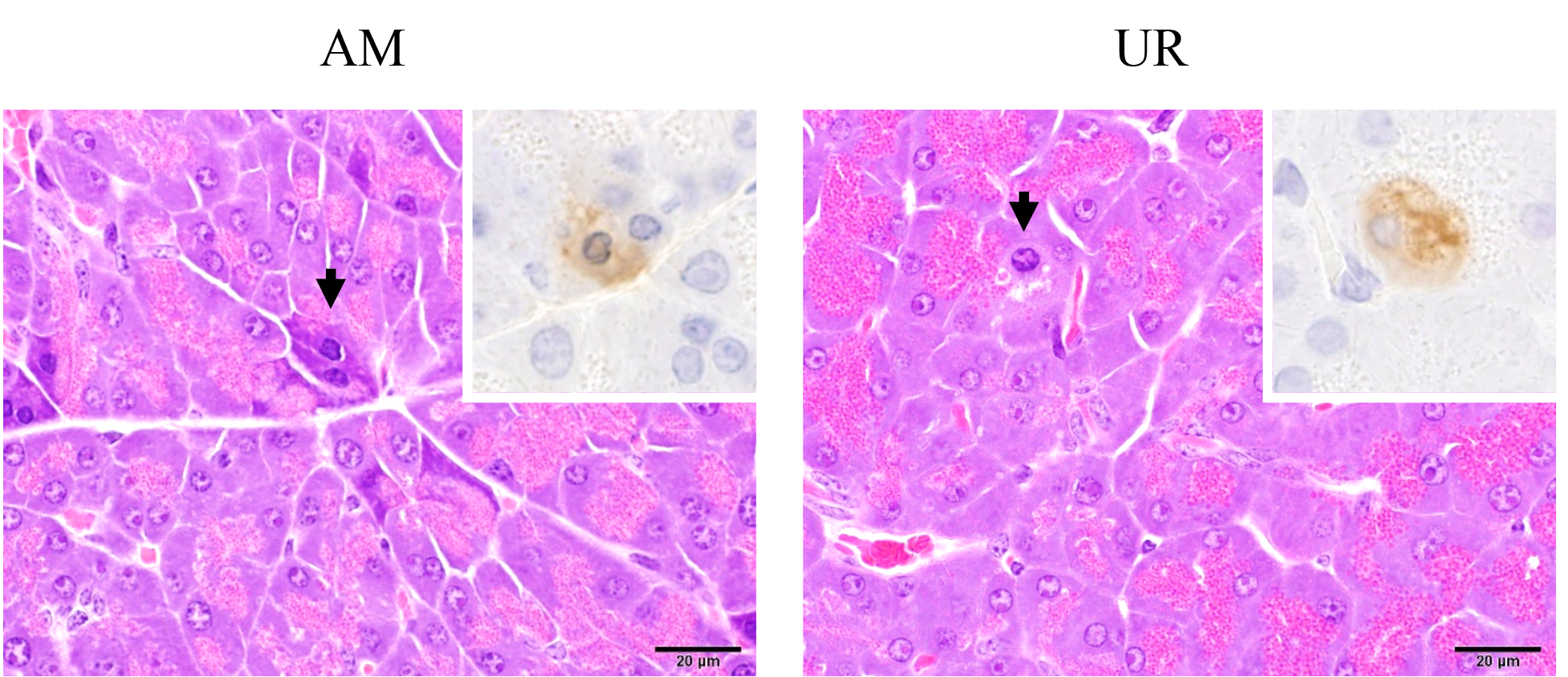

Supplement: S8 Fig — On Day 3 post-inoculation (p.i.), pancreata were obtained from BALB/c mice after intraperitoneal inoculation with 104 CCID50 (cell culture infectious dose) of the aseptic meningitis (AM) and upper respiratory (UR) strains of SAFV-3. Hematoxylin and eosin (H&E) staining and immunohistochemical analysis with an anti-SAFV-3 antibody (anti-SAFV). Bar, 20 μm. Viral antigen-positive cells were seen in acinar cells of the pancreas from both AM- and UR- inoculated mice. The viral antigen-positive cells had a basophilic cytoplasm (arrows). Pancreatic islet cells were negative for viral antigens. Original magnification, 1,000×. (TIF) [file pone.0148184.s008.tif]
