# Supplementary material for: Neuropathogenicity of Two Saffold Virus Type 3 Isolates in Mouse Models
Source: PLoS One. 2016 Feb 1;11(2):e0148184. doi: 10.1371/journal.pone.0148184 (PMC4734772; doi:10.1371/journal.pone.0148184)
Supplement: S1 Table — (DOCX) [file pone.0148184.s009.docx]

**S1 Table.** Histopathological data of neonatal ddY mice after intraperitoneal inoculation with Saffold virus

| Virus strain | Day of sacrifice (p.i.) | Number of mice | Central nervous system tissues | | | |  | Other tissues | | | |
| --- | --- | --- | --- | --- | --- | --- | --- | --- | --- | --- | --- |
|  |  |  | Cerebrum | Brain stem | Cerebellum | Spinal cord |  | Muscle | Pancreas | Oral mucosa | Tooth germ |
| AM | 3 | 4 | 0/0* | 0/0 | 3/0 | 0/0 |  | 2/0 | 0/0 | 0/0 | 0/0 |
|  | 7 | 4 | 2/0 | 0/0 | 1/0 | 0/0 |  | 3/0 | 0/0 | 0/0 | 0/0 |
|  | 19 | 4 | 0/0 | 0/0 | 0/0 | 1/1 |  | 0/0 | 0/0 | 0/0 | 0/0 |
|  |  |  |  |  |  |  |  |  |  |  |  |
| UR | 3 | 4 | 3/0 | 4/0 | 3/0 | 1/0 |  | 4/0 | 0/0 | 0/0 | 1/0 |
|  | 7 | 3 | 3/3 | 2/1 | 2/0 | 0/0 |  | 2/0 | 0/0 | 0/0 | 2/1 |
|  | 21 | 4 | 0/0 | 0/3 | 0/2 | 0/3 |  | 0/0 | 0/0 | 0/0 | 0/0 |

*Values represent the number of animals positive for viral antigen/number of animals showing evidence of degeneration or inflammatory reactions.

AM, aseptic meningitis; p.i., post-inoculation; UR, upper respiratory
