# Supplementary material for: Neuropathogenicity of Two Saffold Virus Type 3 Isolates in Mouse Models
Source: PLoS One. 2016 Feb 1;11(2):e0148184. doi: 10.1371/journal.pone.0148184 (PMC4734772; doi:10.1371/journal.pone.0148184)
Supplement: S2 Table — (DOCX) [file pone.0148184.s010.docx]

**S2 Table.** Detection of viral genome and viral antigen in the brain of adult BALB/c mice after intracerebral inoculation with Saffold virus

| Virus strain | Day of sacrifice (post-inoculation) | Viral genome in the tissue homogenate by nested RT-PCR (n=3 or 6) |  | Viral genome in the paraffin embedded tissue | | Viral antigen in the paraffin embedded tissue by immunohistochemistry  (n=3 or 6) |
| --- | --- | --- | --- | --- | --- | --- |
|  |  |  |  | Real-time RT-PCR  (n=3 or 6) | *in situ* hybridization  (n=1 or 3) |  |
| AM | 3 | 3^§^ |  | 1 | 1** | 3 |
|  | 8 | 3 |  | 0 | 0** | 3 |
|  | 21 | 3 |  | 0 | 0** | 0 |
|  | 60 | 1* |  | 0* | 0** | 0* |
|  |  |  |  |  |  |  |
| UR | 3 | 3 |  | 2 | 1** | 3 |
|  | 8 | 3 |  | 1 | 0** | 3 |
|  | 21 | 3 |  | 0 | 0** | 0 |
|  | 60 | 3* |  | 0* | 0 | 0* |

^§^ Number of positive animals for viral genome or viral antigen

*n=6; **n=1, Examined the positive tissues for viral genome by real-time RT-PCR and/or by nested RT-PCR

AM, aseptic meningitis; UR, upper respiratory
